# Structural and functional insights into the p160 Rho-associated coiled-coil-containing protein kinase ROCK

**DOI:** 10.1038/s42004-026-02258-2

**Published:** 2026-11-04

**Authors:** Badri Nath Dubey, Soheila Rezaei Adariani, Lothar Gremer, Radovan Dvorsky, Yan Nie, Ehsan Amin, Jens M. Moll, Sicai Zhang, Sander H. J. Smits, Melissa A. Graewert, Dieter Willbold, Georg Groth, Luitgard Nagel, Sabrina Pospich, Mohammad R. Ahmadian

**Affiliations:** 1 Institute of Biochemistry and Molecular Biology II, Medical Faculty and University Hospital Düsseldorf, Heinrich Heine University, Düsseldorf, Germany; 2 Department of Biosciences and Bioengineering, Indian Institute of Technology Dharwad, Dharwad, India; 3 Institute of Physical Biology, Heinrich Heine University, Düsseldorf, Germany; 4 Structural Biochemistry (IBI-7), Institute of Biological Information Processing, Research Centre Jülich, Jülich, Germany; 5 Department of Structural Biochemistry, Max Planck Institute of Molecular Physiology, Dortmund, Germany; 6 Institute of Biochemistry and Center for Structural Studies, Heinrich Heine University, Düsseldorf, Germany; 7 European Molecular Biology Laboratory, Hamburg, Germany; 8 Institute of Biochemical Plant Physiology, Heinrich Heine University, Düsseldorf, Germany; 9 Present Address: Department of Neurosurgery, City of Hope Beckman Research Institute and Medical Center, Duarte, CA USA; 10 Present Address: Shanghai Institute for Advanced Immunochemical Studies, ShanghaiTech University, Shanghai, China; 11 Present Address: Institute of Future Agriculture, Northwest Agriculture and Forestry University, Yangling, China

**Keywords:** Structural biology, Electron microscopy

## Abstract

Rho-associated protein kinases (ROCK1/2) are critical regulators of fundamental cellular processes and represent promising therapeutic targets for various pathologies, particularly cardiovascular disease. While the terminal globular kinase domain and the PH-C1 tandem (PHn-C1-PHc) are well characterized, the sequence-structure-function relationship of the central amphipathic α-helical segment remains poorly understood. Utilizing an integrative biophysical approach—including multi-angle light scattering (MALS), analytical ultracentrifugation (AUC), small-angle X-ray scattering (SAXS), negative-stain electron microscopy (EM), X-ray crystallography, and 3D molecular modeling—we elucidate the structural organization of the central coiled-coil region of ROCK1. Notably, the 2.2 Å resolution crystal structure of the homology region 1 (HR1) of ROCK1 reveals a canonical parallel coiled-coil dimer. This structural arrangement diverges significantly from the antiparallel coiled-coil HR1 module found in other Rho effector kinases. Combined with the structural reconstitution of full-length ROCK1, our data demonstrates that the central segment of ROCK1 forms a highly elongated coiled-coil dimer. Furthermore, EM analysis of the isolated full coiled-coil domain uncovers a dynamic oligomerization state, transitioning between a dimer and a tetramer. Together, these biophysical insights establish a foundation that opens avenues for future investigation into ROCK1 regulation, activation, and downstream signaling mechanisms.

## Introduction

The Rho-associated coiled-coil protein kinases (ROCK1/ROKβ/p160ROCK and ROCK2/ROKα/Rho kinase) have been identified as downstream effectors of the Rho isoforms of the Rho protein family^1–8^. These kinases play a significant role in Rho-mediated, stress-induced rearrangements of the actomyosin cytoskeleton at various adhesion sites of different cell types. This process regulates cellular contraction, motility, morphology, polarity, cell division, and gene expression^9–12^.

Emerging evidence suggests that ROCK proteins are involved in different stages of cardiovascular disease, including cerebral and coronary vasospasm, angina, hypertension, pulmonary hypertension, and heart failure^12–14^. This establishes ROCK proteins as an important therapeutic target in cardiovascular medicine^15,16^.

**Fig. 1:**
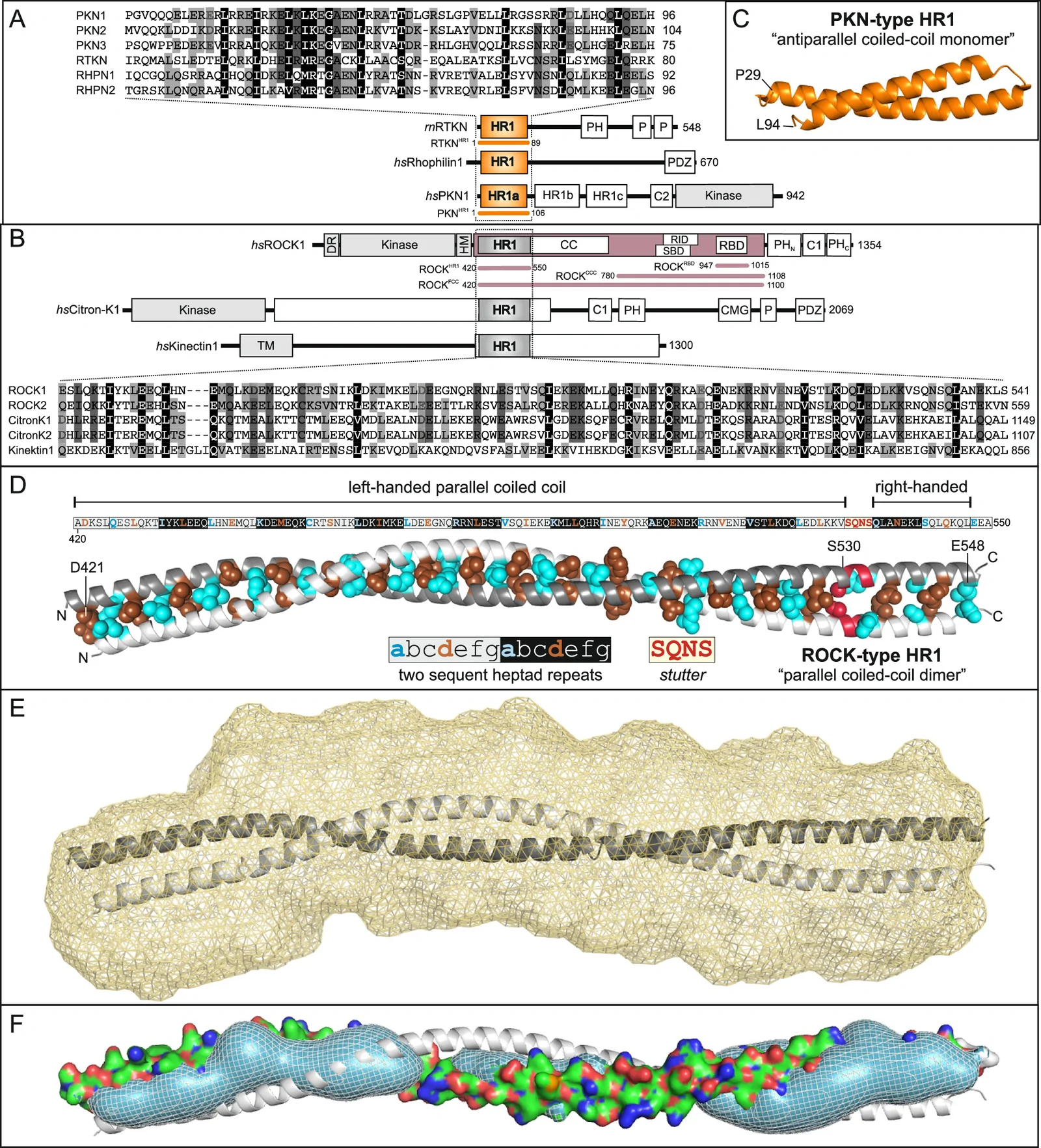
Sequence-structure relationships of ROCK-type and PKN-type HR1 domains. **A**, **B** Show domain organization and sequence comparisons of HR1-domain-containing proteins and the proteins used in this study. The sequence alignment of various HR1-like domains was generated using ClustalW and edited using GeneDoc. Residue shading was performed based on sequence identity and/or similarity criteria. Amino acids with similar properties are shown with a black background. Those with 50–80% homology are shown in dark gray. Amino acids with 30–50% homology are shown with a light gray background. The purified ROCK, PKN, and RTKN proteins used in this study are marked with bold lines and indicated by N- and C-terminal amino acid numbers. C1: Cysteine-rich diacylglycerol binding region; CC: Coiled-coil; CCC: C-terminal coiled-coil; CMG: Citron/MRCK/Gek (profilin IIa binding); DR: Dimerization region; FCC: Full coiled coil; HM: Hydrophobic motif; HR1: Homology region 1; Kinase: Kinase domain; P: Proline-rich; PDZ: Postsynaptic density-95, disc large, and zonula occludens-1; PH: Pleckstrin homology; PHN: N-terminal pleckstrin homology; PHC: C-terminal pleckstrin homology; RBD: RhoA binding domain; RID: RhoA interacting domain; SBD: Shroom binding domain; TM: Transmembrane region; hs: Homo sapiens; rn: Rattus norvegicus; bt: Bos taurus. **C** PKNHR1a forms an antiparallel coiled-coil monomer^28^. **D** The crystal structure of ROCK^HR1^ revealed a parallel coiled-coil dimer with canonical heptad repeats. A cartoon representation of the coiled-coil backbone (in white and gray) and a sphere representation of residues that interact within the heptad repeat frame at positions “a” (in blue) and “d” (in brown) are shown with the sequence of ROCK^HR1^ with heptad repeats (in gray and black backgrounds). The lines above the ROCK^HR1^ structure indicate where the helices twist in a canonical left-handed fashion and where they twist in a non-canonical right-handed fashion after the stutter (in red). **E** The averaged ab initio SAXS shape of ROCK^HR1^ was generated by 20 independent DAMMIF runs and superimposed with the crystal structure of ROCK^HR1^. **F** Distribution and properties of amino acids in ROCK^HR1^. Hydrophobicity is displayed as green lobes around one helix and as a surface colored according to underlying side-chain atoms (red for oxygen, blue for nitrogen, green for carbon; backbone atoms are gray). The lobes of hydrophobic potential (cyan) illustrate the influence of apolar residues, which primarily promote interhelical interactions. This correlates well with the twinning of the second helix.

The highly homologous ROCK proteins share a serine/threonine kinase domain at their N-terminus, followed by a central amphipathic α-helical segment (hereafter called the full coiled coil, or ROCK^FCC^, amino acids 420–1100). At their C-terminus, they have a pleckstrin homology (PH) domain split by an atypical C1 domain (PHn-C1-PHc) (Fig. 1)^10,17^. The insertion of the C1 domain has been shown not to alter the structure of the PH domain, which can bind 3′-phosphate-phosphoinositides^17^. ROCK^FCC^ contains an N-terminal homology region 1 (ROCK^HR1^) domain, a Rho-interacting domain (RID), and a Rho-binding domain (RBD)^7^. The RID overlaps with the shroom-binding domain (SBD), which mediates the formation of the ROCK-shroom complex and regulates cell morphology^18^. Previous fluorescence-based binding assays have demonstrated that the HR1 domain of ROCK binds to RhoA. This finding confirms a direct interaction, which is consistent with the regulatory role of HR1 domains observed in other Rho-associated kinases^7^. HR1 domains (HR1a-c) were first described in protein kinase N1 (PKN1), also known as protein kinase C-related kinase (PRK1), another Rho effector^19^. The HR1 domains of PKN1 have been reported to interact with active forms of RhoA and Rac^20–22^. HR1 domains have also been described in other Rho effectors, such as Rhotekin (RTKN) and Rhophilin-1 (RHPN1). Unlike PKN, these proteins contain only one HR1 domain^20,23,24^. Other Rho effector proteins, such as Citron kinase and Kinectin, also contain a single HR1 domain, similar to ROCK^7^. Unlike the intermolecular parallel coiled-coil structure of ROCK^RBD^ (aa 998–1010)^6,25^ and ROCK^CC^ (aa 535–700)^26^, the structure of ROCK^HR1^ was previously proposed to form an intramolecular antiparallel coiled-coil^7^ in analogy to PKN1^HR1^ structures^22,27,28^. Initial models proposed that ROCK activation occurs through the relief of autoinhibition when Rho binds to its C-terminal region^7,29,30^. However, subsequent structural studies challenged this mechanism. These studies proposed that ROCK exists in a constitutively extended conformation. This places its kinase domain far from the plasma membrane. This raises questions about how Rho engagement promotes myosin activation^8,31^. A recent study revealed an unrecognized interaction between active Rho and the C-terminal PH–C1 tandem domain of ROCK1^32^. This interaction drives the rapid recruitment of full-length ROCKI to sites of Rho activity. Mutational analysis and AlphaFold predictions identified key residues that mediate this interaction. These findings reveal that the PH–C1 domain contributes to ROCKI membrane recruitment, yet it unexpectedly attenuates ROCKI’s ability to activate Myosin II^32^.

In this study, we utilized an integrative biophysical approach to systematically investigate the sequence-structure-property relationship of ROCK1 (hereafter referred to as ROCK). Through a comprehensive sequence analysis of all HR1 domains of Rho effectors, we demonstrate that RhoA effectors partition into two distinct structural classes: the PKN-type and the ROCK-type HR1 domains. Our 2.2 Å resolution crystal structure of ROCK^HR1^ reveals that, contrary to previous assumptions, the ROCK-type HR1 module forms a bona fide parallel coiled-coil dimer. Integrated with biophysical data from additional ROCK fragments covering the central coiled-coil segment, these findings enabled us to reconstruct a comprehensive structural model of the full-length ROCK (ROCK^FL^).

## Materials and methods

### Constructs

The HR1 domains of human ROCK (ROCK^HR1^, aa 420–550), human PKN1 (PKN^HR1^, aa 1–106), rat RTKN (aa 1–89, which is 100% identical to human RTKN^HR1^), ROCK^RBD^ (aa 947–1015), ROCK^CCC^ (C-terminal coiled-coil; aa 780–1108), RhoA (aa 1–193), and MYPT1 full-length (1–1030) were cloned into a pGEX-4T1 vector for expression in *Escherichia coli*, as previously described^7^. ROCK^KD^ (aa 1–420), ROCK^FCC^ (aa 420–1100) and ROCK^FL^ were cloned into a pFastBac HTb vector (Invitrogen) containing an N-terminal His_6_ tag for expression in an insect cell baculovirus system.

### Protein purification

All proteins, except for ROCK^FCC^ and ROCK^FL^, were produced in *E. coli* as described^7^. The Seleno-methionine (Se-Met)-labeled ROCK^HR1^ was obtained in M9 minimal medium containing Se-Met (50 mg/l). Baculoviruses, encoding ROCK^FCC^ and ROCK^FL^, were amplified in *Spodoptera frugiperda* 9 (*Sf*9) and later used to transduce BTI-*Tn*ao38 cells, derived from *Trichoplusia ni*^33,34^ (1.5 × 10^6^ cells/ml) in a culture volume of 2 L. At 4 days post-infection, cells were collected by centrifugation (5000 rpm, 10 min, 4 °C), washed twice in 40 mM HEPES (pH 7.5), 150 mM NaCl, 3 mM β-mercaptoethanol, and 100 µl DNase I, followed by the addition of one protease inhibitor cocktail tablet (EDTA-free, Roche). After cell lysis by sonication and lysate centrifugation (16,000 rpm, 45 min, 4 °C), the supernatant was loaded on a Ni^2+^-NTA column (Qiagen). His-ROCK^FCC^ and ROCK^FL^ were eluted in a buffer containing 40 mM HEPES (pH 7.5), 150 mM NaCl, 3 mM β-mercaptoethanol and 500 mM imidazole and reloaded on a Superdex 200 16/60 column (GE Healthcare) using a buffer containing 40 mM HEPES (pH 7.5), 150 mM NaCl and 3 mM dithiothreitol (DTT). All proteins were snap-frozen in liquid nitrogen and stored at −80 °C.

### Far-UV circular dichroism (CD)

Far-UV CD spectra were measured as described^35^. CD measurements were performed at room temperature for all purified effector proteins using a CD spectropolarimeter. Spectra were recorded in 10 mm path-length cylindrical quartz cuvettes. Wavelength scans were acquired in the far-UV region by averaging ten consecutive spectra at 1 nm intervals, with a scan speed of 200 nm/min. All measurements were carried out in buffer containing 30 mM sodium phosphate (pH 7.5 or 8.5) and 100 mM NaCl, with protein concentrations ranging from 0.2 to 0.5 mg/ml.

### Analytical size exclusion chromatography (SEC)-multi-angle light scattering (MALS)

SEC–MALS experiments were performed as described^36^. For the SEC–MALS experiments, protein samples were injected at a concentration of 0.5 mg/mL in a total volume of 100 µL. Measurements were performed using a miniDAWN multi-angle light scattering detector (Wyatt Technology), which was coupled in-line to a size-exclusion chromatography (SEC) column and an Optilab refractive index detector. Protein samples were separated by gel filtration prior to light scattering detection to ensure monodispersity. Data acquisition and analysis were carried out using ASTRA software (Wyatt Technology), which was also used to calculate molecular weights of the proteins and assess their oligomerization state.

### Analytical ultracentrifugation (AUC)

Sedimentation velocity (SV) centrifugation was performed on a ProteomLab™ XL-A analytical ultracentrifuge (Beckman-Coulter, Brea, CA, USA) using an AN60 Ti rotor with standard aluminum 2-channel centerpieces with quartz windows. The samples were spun at speeds ranging from 35,000 to 50,000 rpm, depending on the protein size, at 20 °C. Radial scans were recorded with 30 µm radial resolution at ~3 min intervals. The software packages UltraScan II v 9.9 and SEDFIT v 14.14 were used for data evaluation. After the transformation of the recorded sedimentation velocity data, taken in the intensity mode to either absorbance or pseudo-absorbance data in the respective data evaluation software, time- as well as radially-invariant noise was calculated and subtracted. In UltraScan, data were fitted as described^37^. In SEDFIT (http://www.analyticalultracentrifugation.com), continuous sedimentation coefficient distributions *c*(*s*) were determined with 0.05 S resolution and an F-ratio = 0.95. Suitable *s*-value ranges between 0 and 30 S, and for GA *f/f*_0_ between 1 and 4 were chosen. Buffer density and viscosity were calculated with SEDNTERP v 20111201 beta (http://bitcwiki.sr.unh.edu/index.php). The partial specific volumes of the studied proteins were calculated according to the method of Cohn and Edsall as implemented in SEDNTERP or UltraScan. Graphical output for SEDFIT results was generated with the software GUSSI v1.0.3, written by Chad A. Brautigam, © 2012 the University of Texas Southwestern Medical Center.

### Sequence and structural analysis

Amino acid sequences were extracted using the multiple sequence alignment programs ClustalW (http://clustalw.genome.ad.jp) and GeneDoc (http://www.nrbsc.org/gfx/genedoc/). Secondary protein structures were predicted using Garnier (http://emboss.bioinformatics.nl). The X-ray structures and full-length ROCK model were visualized using PyMol (https://pymol.org/2/).

### Crystallization and structural determination

Crystals of selenomethionine-substituted ROCK^HR1^ (5–20 mg/ml) were grown by hanging drop vapor diffusion at 18 °C in 23% PEG 3350, 150 mM Tri-potassium citrate and 4% MPD (2-methyl-2,4-pentanediol). The crystal was flash-frozen for data collection and diffracted to a resolution of 2.2 Å. MAD data were collected at 100 K on beamline ID 23-1 at ESRF, Grenoble. The programs iMOSFLM^38^ and XDS^39^ were used for data indexing, integrating, scaling, and for the determination of unit-cell parameters. The crystal belongs to space group C222_1_ with unit-cell parameters *a* = 48.26 Å, *b* = 87.07 Å, *c* = 148.22 Å. The Matthews coefficient suggests the presence of a dimer in an asymmetric unit with an estimated solvent content of 45.0% (v/v)^40^. Initial phases were determined from MAD data at 2.2 Å using three wavelengths (peak 0.97883 Å, inflection 0.97954 Å, and remote 0.96408 Å) with the programs SOLVE and RESOLVE^41^. Five of the eight selenium sites were found with a score of 21.5 and a figure of merit of 0.43. The initial model built by RESOLVE was improved with BUCCANEER^42^ and completed manually using COOT^43^. Refinement was carried out using REFMAC and PHENIX refinement. The final model fitted very well in the electron density with good spatial geometry. 99.6% of residues are located in the appropriate regions of the Ramachandran plot produced by PROCHECK^44^. After final refinement, R_work_ and R_free_ were 21.8% and 28.9%, respectively. The data collection and refinement statistics of the ROCK^HR1^ structure are summarized in Supplementary Table S1.

### SAXS data collection and analysis

SAXS data were collected at the EMBL X33 beamline at the DESY storage ring DORIS III. ROCK^HR1^ samples were adjusted to 18.8 mg/ml, ROCK^FCC^ samples to 19.3–1.45 mg/ml in 30 mM Tris (pH 7.5), 100 mM NaCl, 5 mM MgCl_2_, 3 mM DTT, and measured at room temperature. All data were normalized to the intensity of the transmitted beam, and scattering of the buffer was subtracted. For further processing, the ASTAS software package^45,46^ was utilized. Forward scattering and the radius of gyration were evaluated using the Guinier approximation. For ROCK^FCC^ samples, forward scattering intensities of samples at 19.3 and 1.45 mg/ml were merged using PRIMUS^46^. Distance distribution functions were calculated using AUTOGNOM^47^. Molecular envelopes were determined from *P*(*r*) functions using DAMMIF^48^. DAMAVER^49^ was subsequently used for averaging bead models. Superpositioning of the ROCK^HR1^ crystal structure onto the SAXS model was achieved in SUPCOMB13^50^.

### Liposome sedimentation

For liposome sedimentation assays, we prepared multilamellar vesicles using one of two methods. In the first method, we used 100 µg of bovine brain lipid extract (Folch fraction types I and III, Sigma). In the second method, we used a defined mixture of synthetic lipids (Echelon Biosciences), which we combined at the following weight ratios: 37% phosphatidylserine (PS), 20% phosphatidic acid (PA), 2% NBD-labeled phosphatidylethanolamine (NBD-PE), 10.5% phosphatidylethanolamine (PE), 20% phosphatidylcholine (PC), and a phosphoinositide mix comprising 2% PIP₃, 2% PI(4,5)P₂, 1.5% PI(3,5)P₂, 1.5% PI(3,4)P₂, 1.5% PI(3,4,5)P₃, and 1% each of PI₃, PI₄, and PI₅. The lipids were dried overnight under a laminar flow hood to form a thin film. The dried film was rehydrated in a buffer solution containing 20 mM HEPES-NaOH (pH 7.4) and 100 mM NaCl. This was followed by three cycles of snap freezing and thawing. Lipid dispersion was further enhanced by 5 min of bath sonication. To produce uniformly sized unilamellar vesicles, the suspension was passed 21 times through a 50-nm polycarbonate filter using an Avanti extruder. For sedimentation assays, purified ROCK^FL^ was incubated with the prepared liposomes for 30 min on ice. The samples were then centrifuged at 20,000 × *g* for 20 min at 4 °C. The supernatant and pellet fractions were collected separately and analyzed by SDS-PAGE, followed by immunoblotting.

### Kinase assay

The ROCK^FL^ activity in the presence or absence of an active RhoA form was measured using the myosin phosphatase target subunit 1 (MYPT1) as the ROCK substrate. The assay contained: 400 µM ATP, 5 µM MYPT1, 0.02 µM ROCK^FL^, 5 µM GppNHp-bound RhoA, and 5 µM ROCK inhibitor Y-27634. GppNHp is a non-hydrolyzable GTP analog. The assay was conducted at the following time points: 0, 5, 15, 30, 60, and 120 min, with a final sample volume of 30 µl. The kinase reaction was stopped by adding 9 µL of Laemmli buffer (5×) and 10 µl of urea (10 M) to the samples, which were then boiled for 5 min at 99 °C. To determine the MYPT1 phosphorylation state, we analysed the samples by immunoblotting using MYPT1 and phospho-MYPT1 (Thr853) antibodies in a 1:1000 dilution (2634S and 4563S, Cell Signaling Technology).

### Negative stain electron microscopy

Sample droplets of 4 µl were adsorbed for 1 min on freshly glow-discharged copper grids (Agar scientific; G2400C) covered by a thin, continuous carbon film. The grids were then negatively stained with 0.75% uranyl formate (Polysciences) for 1 min before blotting with filter paper (Whatman no. 4). Images were taken at a JEOL JEM-1400 or FEI Tecnai G Spirit electron microscope equipped with a LaB_6_ cathode and operated at 120 kV. Digital electron micrographs were recorded with a 4k × 4k CMOS camera F416 (TVIPS). The length and width of 176 individual ROCK^FCC^ filaments from 21 images were measured using a boxer from the EMAN software package^51^.

### Composite structural model of full-length ROCK

A composite structural model of full-length ROCK was generated by integrating experimentally determined domain structures with predicted models for regions lacking structural information. The final model encompassed the entire ROCK polypeptide, extending from the N-terminal kinase domain to the C-terminal pleckstrin homology (PH) domain. Experimentally determined structures were obtained from the Protein Data Bank and used as template structures to assemble a grand final template for homology modelling. These experimental structures in included the kinase domain dimer (PDB 2ESM), ROCK^HR1^ (PDB 8P0S; this study), ROCK^CC^ (PDB 3O0Z), ROCK^SBD^ (PDB 2L4W), ROCK^RBD^ (PDBs 1S1C and 1UIX), and the PHn–C1–PHc region (PDB 2ROV). All structures were used as crystallographic symmetry mates, solvent molecules, and non-essential ligands were removed prior to assembly. First, a canonical coiled-coil structure was generated for the central region of ROCK (residues 423–1011), which is predicted to adopt this fold. Second, all known structures for this region were superposed onto their corresponding parts. Fragments of the canonical core that overlapped with the template structures were then removed. For overlapping template structures, regions closer to the canonical fold were retained while the remaining parts were removed. Additionally, residues from structures that deviated by more than 3.0 Å from the canonical core were excluded. In the third step, the kinase domain, as well as the PH and C1 domains, were manually placed at the N- and C-termini of the coiled-coil template, respectively, using PyMOL. They were placed and oriented in plausible positions to leave sufficient space for the missing loops, thereby creating the complete template. Finally, after renumbering all residues in the template in accordance with the full-length sequence of ROCK, MODELLER was used to generate a homology model of the full-length protein.

The resulting full-length ROCK composite model spanned approximately 100 nm, consistent with dimensions observed by negative-stain electron microscopy. Structural visualization and distance measurements were performed in PyMOL. The overall architecture of the composite model was assessed qualitatively by comparison with negative-stain EM class averages, with which it showed good agreement.

## Results

### ROCK^HR1^ differs from PKN^HR1^

A comprehensive sequence analysis of the RhoA effectors concerning the HR1 domain revealed that there are significant differences in the HR1 domains of RTKN, RHPN1, and PKN (Fig. 1A) compared to the HR1-like domains of Citron, Kinectin, and ROCK (Fig. 1B). The sequence of ROCK-type HR1, hereafter referred to as ROCK^HR1^, is longer and does not share the same homology as compared to that of the PKN-type HR1 (PKN^HR1^) sequence. These differences suggest a fundamental distinction between the two types of HR1, likely related to their structure and/or mode of interaction.

**Fig. 2:**
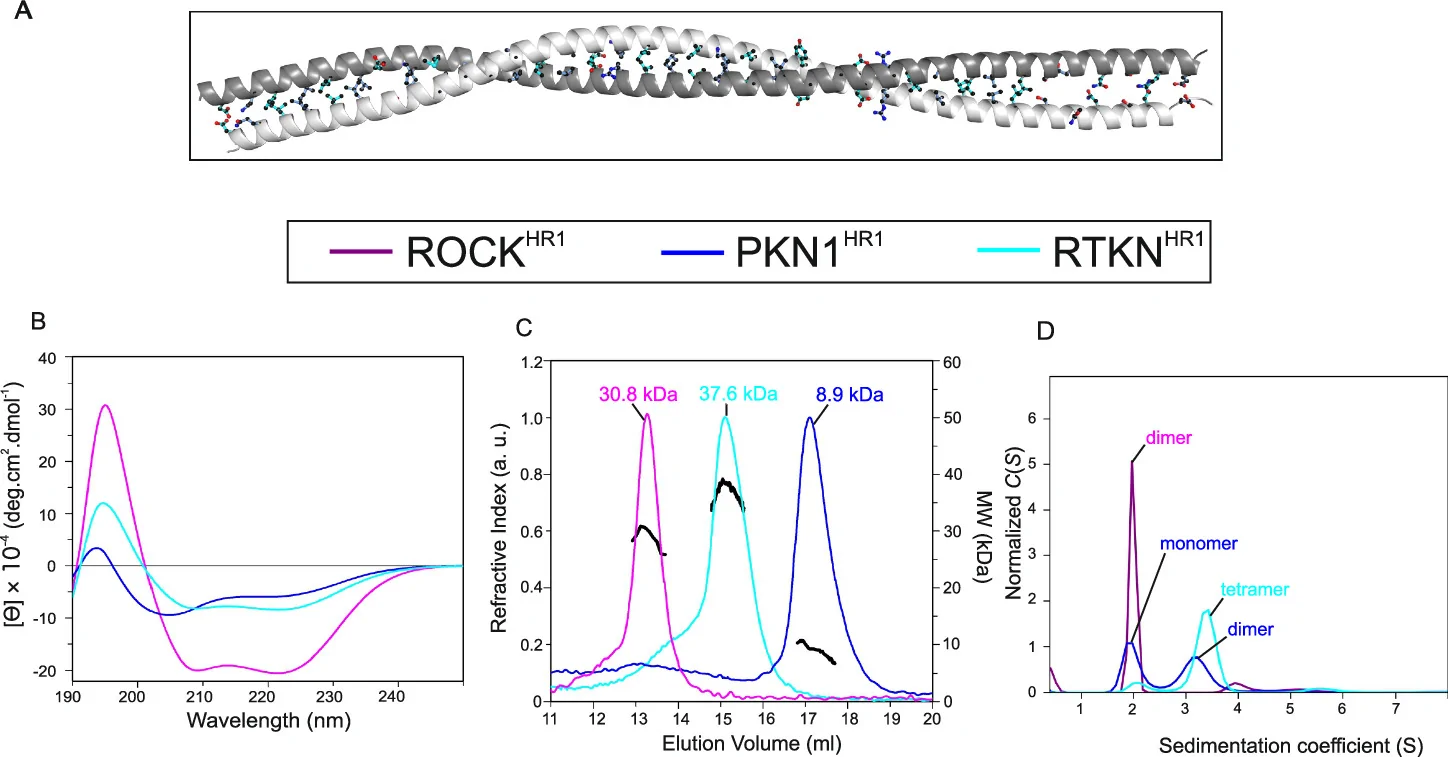
Biophysical properties of the HR1 domains of ROCK, PKN1, and RTKN. **A** The interacting residues are shown as ball-and-stick models between the ribbon helices of the coiled-coil dimer. Atoms are colored as follows: carbon, black; nitrogen, blue; oxygen, red; sulfur, yellow. **B** Far-ultraviolet circular dichroism (Far-UV CD) spectra. The CD data were adjusted for molar ellipticity (Θ), taking into account the concentration and molar mass of the protein samples. Each spectrum is the average of ten replicate scans. The spectra exhibit typical α-helical profiles, displaying a minimum at 208 and 222 nm, except for PKN1^HR1^, which exhibits a minimum at 204 nm instead of 208 nm. **C** Size exclusion chromatography-multiangle light scattering (SEC-MALS) analysis. The protein elution profile by refractive index (RI). The horizontal black line indicates the molar mass of the respective proteins. **D** Sedimentation velocity (SV) analysis. This is a comparison plot of the diffusion-corrected integral sedimentation coefficient distributions obtained from van Holde–Weischet analyses. PKN1^HR1^ is shown in blue, ROCK^HR1^ in magenta, and RTKN^HR1^ in cyan. All data are summarized in Table 2.

In light of these indications, we examined the biophysical and structural characteristics of ROCK^HR1^ (130 aa; calculated molecular weight 15.8 kDa) in direct comparison to the HR1 domains of PKN (PKN^HR1^, 106 aa; 12 kDa) and RTKN (RTKN^HR1^, 89 aa; 10 kDa) (Fig. 1A, B) using different methods, including far-UV CD, MALS, and AUC. The HR1 proteins were purified to high purity (>98%) as judged by Coomassie Brilliant Blue-stained SDS-PAGE gels of the samples used for subsequent biophysical and structural studies. We first analyzed the folding and secondary structure elements of the proteins by far-UV CD spectroscopy. The far-UV CD spectra of these HR1 domains exhibit characteristics typical of α-helical proteins (Fig. 2B). ROCK^HR1^ and RTKN^HR1^ showed negative minima at 208 and 222 nm, respectively. A slight shift of the minimum to 204 nm was observed for PKN^HR1^_,_ indicating that this domain may contain partially unfolded or unstructured regions (Fig. 2B).

Another notable finding was the distinct oligomerization properties exhibited by the three HR1 domains upon elution from a Superdex 200 10/300 SEC column. The estimated molecular weights, estimated from the SEC peaks, were approximately 147 kDa for ROCK^HR1^, 25 kDa for PKN1^HR1^, and 63 kDa for RTKN^HR1^. Since the separation of non-globular coiled-coil proteins by SEC does not correlate with their size, we performed MALS experiments to more precisely assess this relationship. Accordingly, ROCK^HR1^ formed dimers with an apparent molecular mass of 31 kDa (Fig. 2C). In contrast, PKN^HR1^ appeared predominantly monomeric, as previously reported^28^, while RTKN^HR1^ showed a strong tendency to form tetramers (Fig. 2D). As summarized in Table 1, these data suggest that the three HR1 domains adopt diverse quaternary structure arrangements.

**Table 1:** The apparent molecular masses of various Rho effector proteins were determined using multiple-angle light scattering (MALS) and analytical ultracentrifugation (AUC) analyses

Proteins (aa)	MW (kDa)		
	Theoretical^a^	MALS	AUC
ROCK^HR1^ (420–550)	15.8	30.8 (dimer)	31.9 (80%; dimer) 95.7 (14.5%; hexamer)
ROCK^RBD^ (947–1015)	8.4	8.9 (monomer)	16.0 (60.7%; dimer)
ROCK^CCC^ (780–1108)	38.9	81.7 (dimer)	61.1 (83.6%; dimer)
ROCK^FCC^ (420–1100)	80.8	289.9 (tetramer)	181.2 (23.8%; dimer) 341.8 (20.4%; tetramer)
PKN^HR1^ (1–106)	11.8	8.9 (monomer)	11.2 (46%; monomer) 22.4 (51%; dimer)
RTKN^HR1^ (1–89)	10.3	37.6 (tetramer)	40.7 (84%; tetramer)

^a^The theoretical molecular mass of each protein was calculated as a monomer using the ProtParam tool available on the ExPASy Molecular Biology Server (www.espasy.org).

To examine this issue in more detail, we conducted AUC experiments using 44 µM of each HR1 protein (Table 1 and Fig. 2D). We then evaluated the results using a genetic algorithm and Monte Carlo analysis (Table 1). ROCK^HR1^ was predominantly found in a dimeric state (>84%; Fig. 2D), consistent with MALS analysis. The detection of a minor contribution of larger species of ROCK^HR1^ indicates some degree of oligomerization. PKN^HR1^ equally appeared in both monomeric and dimeric states, suggesting that the chosen concentration was within the dissociation constant (*K*_d_) range of the dimer (Fig. 2D). The similar *S*-values of ROCK^HR1^, RTKN^HR1^, and PKN1^HR1^ arise because the elongated coiled-coil structure of ROCK^HR1^ and RTKN^HR1^ increases their frictional coefficients, causing the larger ROCK^HR1^ dimer (~31 kDa) and RTKN^HR1^ tetramer (~40 kDa) to sediment at rates comparable to the smaller, more flexible PKN1^HR1^ monomer (~12 kDa) and dimer (~24 kDa), respectively. Based on this observation, an equilibrium *K*_d_ of 44 µM was estimated for the PKN^HR1^ homodimerization. For RTKN^HR1^, two solution forms, consistent with dimeric and tetrameric assemblies, were observed with the tetrameric form being the predominant species (>80%) (Fig. 2D and Table 1). The CD spectrum of PKN1^HR1^ (1–106) exhibits a distinct profile. Notably, there are fewer minima at 220 nm than in the spectra of ROCK^HR1^ and RTKN^HR1^. This suggests a partially unfolded conformational state. SEC-MALS analysis yielded a molecular mass of 8.9 kDa for PKN1^HR1^, lower than the theoretical monomeric mass of 11.8 kDa (Fig. 2 and Table 1). SEC-MALS provides an absolute molecular-mass measurement that is independent of the protein’s globular shape or conformational state. AUC analysis revealed a heterogeneous population comprising 46% monomer (11.2 kDa) and 51% dimer (22.4 kDa). Although PKN1^HR1^ predominantly behaves as an undersized monomer in SEC-MALS experiments, these results suggest that it retains a limited propensity for self-association under sedimentation conditions. The lower-than-expected molecular mass measured by SEC-MALS is potentially arising from partially degraded or truncated species, resulting in a lower mass averaged across the eluting population. In contrast to ROCK^HR1^ and RTKN^HR1^, the molar-mass trace of PKN1^HR1^ exhibited a pronounced downward slope across the elution peak, indicating molecular heterogeneity (Fig. 2B). Given the lower-than-expected molecular mass, this heterogeneity may reflect the presence of full-length protein together with partially degraded or truncated species. Therefore, the SEC-MALS-derived mass for PKN1^HR1^ represents the molecular mass averaged across the analysed eluting population rather than that of a single discrete molecular species. Taken together, the SEC-MALS, AUC, and CD data provide convergent evidence that PKN1^HR1^ does not adopt a stable, fully folded coiled-coil conformation under the tested experimental conditions. This contrasts with the well-ordered dimeric and tetrameric assemblies observed for ROCK^HR1^ and RTKN^HR1^, respectively.

Taken together, our biophysical data demonstrate that ROCK^HR1^ is a stable dimer. This contrasts with PKN^HR1^, which exists in monomeric and dimeric forms, and RTKN^HR1^, which forms higher oligomers. These data reveal unexpected structural variations within the HR1 domains of ROCK- and PKN-type sequences. ROCK-type HR1 domains form stable dimers, while PKN-type HR1 domains allow for more structural variability due to their sequences.

### ROCK^HR1^ is a bona fide parallel coiled-coil dimer

Our observations that ROCK^HR1^ and PKN^HR1^ belong to two different classes of RhoA effector kinases, based on their sequences and the existence of different oligomeric states, were highly intriguing. Therefore, we set out to investigate the structure of ROCK^HR1^ using X-ray crystallography. Complete data sets of ROCK^HR1(Se-Met)^ crystals were collected at the European Synchrotron Radiation Facility (ESRF). The structure of the ROCK^HR1^ dimer complex within the asymmetric unit of the crystal was determined using multi-wavelength anomalous dispersion (MAD) and refined to a resolution of 2.2 Å (Supplementary Table S1).

Unlike the antiparallel coiled-coil structure of PKN^HR1^ (Fig. 1C)^22,28^, the structure of ROCK^HR1^ consists of two long α-helices (188 Å) that intertwine to form a parallel, two-stranded coiled-coil dimer (Fig. 1D). The total buried solvent accessible surface area at the coiled-coil interface is 1926 Å^2^. ROCK^HR1^ largely exhibits the characteristic features of a canonical left-handed coiled-coil with a periodicity of seven residues over two helical turns, known as a heptad repeat (Fig. 1D). Each heptad repeat in ROCK^HR1^ extends the length of the coiled-coil by ~1 nm, resulting in a total length of 19.5 nm.

Additional insights into the arrangement of ROCK^HR1^ molecules in solution were obtained through SAXS analyses. The geometric properties of the ROCK^HR1^ molecules were estimated using Guinier analysis and their *P*(*r*) functions. These analyses yielded the following values: a reciprocal space radius of gyration (Rg^rec^) of 41 ± 1.1 Å, a real space radius of gyration (Rg^real^) of 48 ± 0.1 Å, and a maximum particle diameter (*D*_max_) of 165 Å. From the *P*(*r*) function, a total of 20 molecular envelopes were calculated for ROCK^HR1^ and subsequently averaged (Supplementary Fig. S2). The final averaged model resembles a rod-shaped particle, which is consistent with the coiled-coil structure of ROCK^HR1^ (Supplementary Fig. S2A, B). Superposing the crystal structure onto the SAXS envelope revealed a perfect match between the results obtained by the two different techniques (Fig. 1E). Additionally, a theoretical scattering curve was calculated from the ROCK^HR1^ crystal structure. This scattering curve could in turn be aligned with the SAXS data, providing a reasonable fit (*χ*^2^ = 3.2) (Supplementary Fig. S2A). Taken together, these data support the findings provided by X-ray crystallography, which propose that ROCK^HR1^ adopts an elongated coiled-coil assembly in solution.

Using the TWISTER program^52^, we identified seventeen heptad repeats within the coiled-coil region of ROCK^HR1^, spanning from Q425 and L548. An insertion of four amino acids (SQNS) at the positions 530–533, called “stutter,”^53^ caused a transition from a left-handed to a right-handed coiled-coil arrangement. The heptad-repeat pattern identified in the ROCK^HR1^ structure (Fig. 1D) aligns with the pattern predicted from the ROCK sequence using the COIL^54^ program. The stutter found at the C-terminus of ROCK^HR1^ also corresponds to the predicted interruption of repeats. The only difference is that the two repeats after the stutter do not correspond to the predicted repeats; they are shifted by one residue and form a non-canonical right-handed coiled-coil. These two predicted heptads, however, form a coiled-coil in the structure, but between the C-termini of symmetry-related molecules and remarkably in an antiparallel fashion.

### The surface area of the ROCK^HR1^ dimer displays amphipathic characteristics

The amphipathicity of a coiled-coil is defined by a dual character of the amino acids on the surface of the involved α-helices. Accordingly, at the interface between two helices alongside the coiled-coil, there should be amino acids with apolar side chains, and polar residues should be outside of that interface. Since classical mapping of the amino acid polarity on the molecular solvent-accessible surfaces could not clearly demonstrate the amphipathic feature of the ROCK^HR1^ coiled-coil, we have calculated the pseudo-hydrophobic potential around one of its helices. As shown in Fig. 1F, lobes of hydrophobic potential, which indicate favorable interaction with apolar residues at this place, correlate perfectly with the twinning of the second helix (see “Methods”). The same would be true at the C-terminus if the coiled-coil twist were not broken. Consequently, polar residues evoking no hydrophobic potential are located outside of the interface between the two helices. The exception of this scenario is the region between the residues 498 and 509, where three of the contacting residues of the helices are not hydrophobic, namely Y498, E505, and R509. Another descriptor of coiled-coil geometry is its radius, which is 4.8 Å for an ideal coiled-coil. The radii of ROCK^HR1^ mostly oscillate around this value with a large deviation at the C-terminus, presumably caused by its non-canonical fold (Supplementary Fig. S3A). However, a “swell” exists between residues 440 and 450 that could not be directly explained. For this reason, we set out to explore the interaction between amino acids in detail (Supplementary Fig. S1).

### The ROCK^HR1^ structure exhibits different atypical heptad repeats

Consistent with previous observations, half of the amino acid contacts exhibit classical knobs-into-holes packing (Supplementary Fig. S1). Regarding the other interactions between the two helices at positions “a” and “d” in the heptad repeats, the vast majority of them are hydrophobic (Supplementary Fig. S1A, B). Several interactions between helices are mediated by polar residues that face each other with their hydrophobic side-chain parts, similar to knobs-into-holes interactions (Supplementary Fig. S1B). The increased radius of the coiled-coil between the residues 440 and 450 (Supplementary Fig. S3A) appears to be caused by two atypical coiled-coil interactions, namely E442/E442′ and K446/K446′, as well as contacts between M449/M449′. These contacts cannot be packed ergonomically within the coiled-coil (Supplementary Fig. S1C, D). Notably, five out of the six polar interactions comprise the C-terminus after the stutter, where the coiled-coil fold is not canonical (Supplementary Fig. S1E). Residues outside of the interacting interface of the coiled-coil are predominantly hydrophilic/polar except at the C-terminus, where the coiled-coil fold breaks. In addition, there is no correlation between the amino acid conservation and their occurrence alongside the ROCK^HR1^.

### The highly elongated ROCK^FCC^ switches between a dimer and a tetramer

**Fig. 3:**
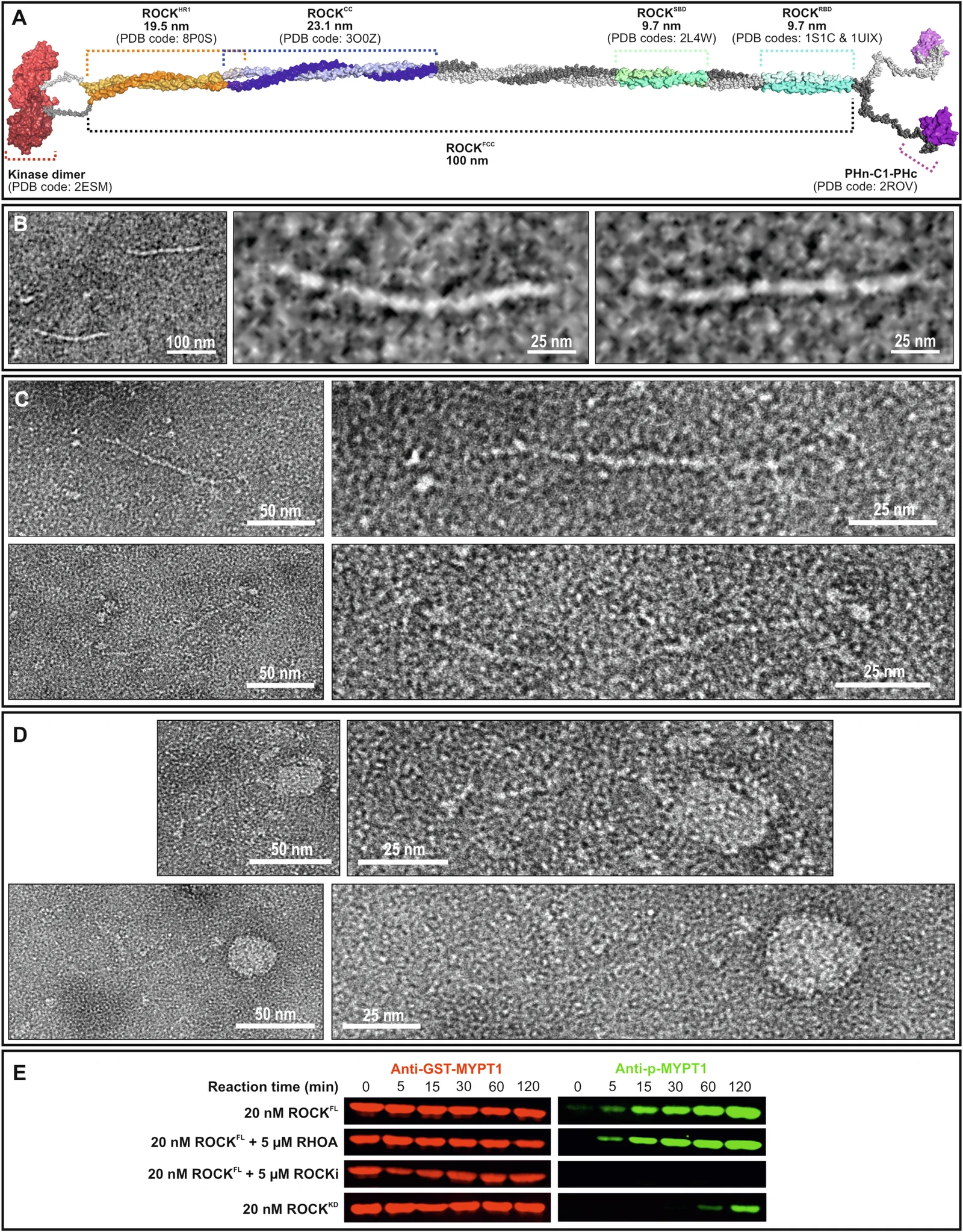
Structural properties of ROCK. **A** A structural model of dimeric ROCK^FL^. The ROCK structure was assembled from various available structures of different regions, including the kinase domain (red^55^), ROCK^HR1^ (orange; this study), ROCK^CC^ (blue^26^), ROCK-RBD (turquoise^5,25^, the PHn-C1-PHc (purple^17^) as indicated by the protein database (PDB) codes. Regions in gray were modeled due to the lack of available structural data. A tentative assignment of the terminal kinase and the PHn-C1-PHc domains is highlighted in two different colors. These domains are separated by an estimated 100 nm long, elongated coiled-coil region. **B**–**D** Electron micrographs of negatively stained ROCK^FCC^ and ROCK^FL^. Representative EM images of negatively stained ROCK^FCC^ (**B**) and ROCK^FL^ (**C**), respectively, reveal an elongated and filament-like structure of ROCK, with enlarged structures displayed in the right panels. The scale bars are indicated within each image. **E** ROCK kinase assay. The phosphorylation of MYPT1 by ROCK^FL^ in both the absence and presence of GppNHp-bound RhoA was examined at various time intervals and at a temperature of 25 °C. Proteins were purified from the insect cells and *E. coli*. The samples were analyzed by Western blotting using antibodies against GST-MYPT1 and p-MYPT1. As controls, the experiments were performed in the absence of ROCK^FL^, and with ROCK^KD^, and in the presence of the ROCK inhibitor Y-27632, all under the same conditions.

The central amphipathic region of ROCK (called ROCK^FCC^) contains the HR1 and RBD domains at its N- and C-termini, respectively. These domains, together with the structures of both ROCK^CC^ (aa 535–700; immediately downstream of ROCK^HR1^) and ROCK^SBD^ (aa 834–913; overlapping with ROCK^RID^; Fig. 1B), form a parallel α-helical coiled-coil dimer. These coiled-coils, together with the terminal globular structures, including the N-terminal kinase domain and the C-terminal split PH-C1 tandem, PHn-C1-PHc^17,55^ provide the basis for the ROCK^FL^ structure reconstitution that we performed in a stepwise manner using molecular modeling (see “Methods” for details). Most remarkably, this model revealed that ROCK^FCC^ adopts a 100 nm extended coiled-coil dimer that ends with the functionally conserved globular structures of the kinase and PH-C1 domains (Fig. 3A). This observation raised important questions about the nature and the physical properties of the central coiled-coil region of ROCK^FCC^, which we investigated further. To gain additional insights into the sequence-structure relationship of ROCK^FCC^, we inspected the primary and secondary structures of both ROCKI and ROCKII by performing multiple sequence analyses and secondary structure predictions using Garnier (http://emboss.bioinformatics.nl). We found that both proteins contain more than 90% α-helices, and that approximately 10% of the sequences were predicted to be random coils. Next, we generated various additional ROCK fragments, including ROCK^FCC^ (680 aa; 81 kDa), ROCK^CCC^ (328 aa; 39 kDa) and ROCK^RBD^ (68 aa; 8.4 kDa) (Fig. 1 and Table 1). The corresponding proteins were purified from insect cells (ROCK^FCC^) and *E. coli* (ROCK^CCC^ and ROCK^RBD^). The far-UV CD spectra of these proteins, obtained under the same conditions as described for the HR1 domains, showed characteristics of typical α-helical proteins. These spectra showed a minimum at 208 nm and a maximum at 222 nm, which are consistent with secondary structure predictions (Supplementary Fig. S4A). However, an exception was observed for ROCK^RBD^, which exhibited a slight shift of the minimum from 208 to 204 nm. This shift may be due to partially unfolded or unstructured regions.

To gain insight into the oligomerization state of these ROCK fragments, we performed SEC-MALS and AUC experiments. Using SEC-MALS, we observed increasing oligomerization with increasing protein size, namely ROCK^RBD^ as a monomer, ROCK^CCC^ as a dimer, and ROCK^FCC^ as a tetramer (Supplementary Fig. S4B and Table 1). ROCK^FCC^ exhibited two small shoulders within the main peak, suggesting sample heterogeneity (Supplementary Fig. S4B). The AUC data revealed that ROCK^FCC^ distributes into dimers (60%), tetramers (16%), and also larger aggregates (24%) (Supplementary Fig. S4C and Table 1), whereas ROCK^CCC^ preferentially forms dimers (83%). A minor contribution of larger species (10%) was also detected for ROCK^CCC^ as well, which may be due to larger aggregates. For ROCK^RBD,^ an equal distribution of dimers (24%) and tetramers (20%) was observed.

Next, we conducted SAXS and EM analyses, which are very useful for characterizing the nanoscale ROCK^FCC^ architecture. The SAXS forward scattering intensities suggest a shift from the rod-like characteristics towards a more flexible and globular shape in the case of the full amphipathic region (Supplementary Fig. S2). Similar to ROCK^HR1^, we estimated geometric properties from the Guinier analysis and the *P*(*r*) function (Supplementary Fig. S2D). The radii of gyration were determined to be Rg^rec^ = 78 Å ± 3.3 Å and Rg^real^ = 73 Å ± 0.4 Å, with a maximum diameter of *D*_max_ = 239 Å (Supplementary Fig. S2B and Table 2).

**Table 2:** SAXS data collection and analysis of ROCKHR1 and ROCKFCC

Data collection	ROCK^HR1^	ROCK^FCC^
**Data collection**		
Radiation source	X33 (DORIS @ DESY, Germany)	
s-range (nm^−1^)	0.08–6.0	
Exposure time (s)	15	
**Structural parameters**		
*R*_G_ (from Guinier, Å)	41 ± 1.1	78 ± 3.3
*R*_G_ (from Guinier, Å)	48 ± 0.1	73 ± 4.0
*D*_max_ (Å)	165	239
**Software utilized**		
Data reduction and averaging	PRIMUS	
Guinier and *P*(*r*) distribution	GNOM	
Modelling programs	Dammif, Crysol	

### ROCK^FL^ reveals a parallel elongated, filament-like structure

Due to its small size, visualizing ROCK^FCC^ by negative-stain electron microscopy (EM) was challenging. Nevertheless, we successfully identified elongated filament-like structures of various lengths (Fig. 3B). The particles were approximately 3 nm in diameter and measured 20–110 nm in length (Supplementary Fig. S5). The diameter corresponds well with that of the ROCK^FCC^ of the modeled ROCK^FL^ structure (Fig. 3A). However, the length is partially shorter than the expected length for the full-length ROCK^FCC,^ which should measure ~100 nm. This discrepancy may be due to shearing forces that could break the extremely long coiled coil of ROCK^FCC^ during purification.

EM analysis of purified ROCK^FL^ revealed an elongated structure, approximately 120 nm in length, with prominent globular densities of the kinase domains and the PHn-C1-PHc domains at both ends (Fig. 3C). One end of the structure appears as a tight, compact globular entity, whereas the other end is more extended and segmented, with discernible subdomains. This asymmetry could hint that the compact region corresponds to the kinase domain, while the more modular region represents the PHn–C1–PHc assembly (Fig. 3C). This organization is in line with previous high-resolution studies of individual coiled-coil segments and consistent with the structural models (Fig. 3A; PDB: 2L4W, PDB: 1UIX, PDB: 1S1C and PDB:3O0Z) in which ROCK^FL^ forms a parallel dimer via a conventional coiled-coil.

Taken together, these data suggest that the central region of ROCK forms an elongated α-helical coiled-coil dimer, which is flanked by the globular kinase and the PHn-C1-PHc domains.

### Purified ROCK^FL^ exhibits high activity

To examine the enzymatic activity of ROCK^FL^, the natural substrate MYPT1 was purified as a GST fusion protein. Therefore, 5 µM GST-MYPT1 and 400 µM ATP were used for all conditions at different time points. The reaction was stopped by mixing the samples with Laemmli buffer, containing urea, and denaturing them at 95 °C for 5 min. MYPT1 phosphorylation was detected via Western blotting using anti-phospho-MYPT1 (p-MYPT1) and anti-GST antibodies against non-phosphorylated MYPT1. As shown in Fig. 3E, ROCK^FL^ activity was strikingly higher than that of purified ROCK^KD^. The kinase domain contains short N- and C-terminal extensions that have been reported to facilitate head-to-head dimerization of the kinase lobes and thus full functionality^55^. As a negative control, the ROCK inhibitor Y-27634 fully inhibited the ROCK^FL^ activity. However, the addition of active, GppNHp-bound RhoA did not further enhance ROCK^FL^ activity. The levels of MYPT1 phosphorylation are similar between the two samples, ROCK^FL^ with and without RhoA•GppNHp (Fig. 3E). The reduced activity of the kinase-only construct is likely a direct consequence of lacking these regulatory extensions, rather than an inherent property of the catalytic mechanism itself.

The results indicate that the purified ROCK^FL^ protein exhibits high kinase activity toward its substrate, MYPT1. However, the presence of RhoA does not appear to promote this process further under these conditions.

## Discussion

In the present study, we have comprehensively characterized the physical and structural properties of distinct fragments spanning the central amphipathic region of ROCK, namely HR1, CCC, RBD, and FCC (Fig. 1B). By integrating computational bioinformatics with a robust pipeline of biophysical techniques—including far-UV CD, SEC-MALS, AUC, SAXS, EM, and X-ray crystallography—we have mapped the architecture of this signaling scaffold. Crucially, the crystal structure of ROCK^HR1^ revealed that the N-terminal 130 aa of ${{\rm{ROCK}}}^{{{{\rm{FCC}}}}^{{{\rm{HR1}}}}}$ form a parallel coiled-coil dimer, rather than the previously proposed anti-parallel coiled-coil monomer^7^. This unexpected structure prompted a systematic re-evaluation of the adjacent segments within ROCK^FCC^. We found that RBD and CCC exist in a dynamic equilibrium between dimeric and tetrameric states, a behavior supported by the existing structures of RBD^5,25,28^ and SBD^18^. Low-resolution EM structure of human ROCK2 previously demonstrated a constitutive dimer approximately 120 nm in length, featuring a prominent coiled-coil tether connecting the kinase and membrane-binding domains^8,31^. Our analysis of the isolated fragment directly corroborates this model, confirming that it forms a continuous, parallel coiled-coil homodimer capable of concentration-dependent higher-order oligomerization. Together, these findings provide a definitive structural framework for ROCK^FCC^, positioning the N-terminal globular kinase and the C-terminal PHn-C1-PHc domains more than 100 nm apart (Fig. 3A).

A central question addressed by this study is the functional evolutionary benefit of maintaining such an exceptionally long coiled-coil intervening spacer. Coiled coils are highly versatile rod-like structural elements capable of spanning cellular distances while positioning functional domains with precise stoichiometry and orientation^53,56^. The most prominent feature of ROCK^FCC^ oligomeric plasticity, which is oligomeric plasticity^10,17,29,55,57^. Recent in silico modeling suggested that the N-terminal kinase domain^55^ and the C-terminal split PHn-C1-PHc tandem can physically interact^58^, supporting a classical model where the C-terminus loops back to autoinhibit the catalytic head^1,10^. Interestingly, our structural models indicate that the lipid-binding surfaces of the PHn-C1-PHc domains remain fully accessible even in a conformed state. Cooperative membrane bilayer binding occurs via clustered positively charged patches distributed across these domains^17^, and here we demonstrate via electron microscopy that purified ROCK^FL^ robustly associates with biomimetic liposomes.

While the PHn-C1-PHc tandem binds membranes as a module, the kinase domain forms a stable functional dimer^17,29^ driven by its flanking dimerization region (DR; aa 47–78) and a hydrophobic motif (HM; aa 370–420) (Fig. 1B)^29,57,59^. However, previous SEC analysis of rat brain extracts^60^ alongside our current MALS and AUC analyses (Table 1), reveal that ROCK^FL^ can also assemble into tetramers^60^. We observed that ROCK^FCC^ transitions into higher-order oligomers at micromolar concentrations. In the crowded cellular environment, local ROCK concentrations can increase dramatically via macromolecular crowding or scaffold-mediated membrane recruitment. Our AUC data demonstrate that a discrete fraction of ROCK^FCC^ sediments as a defined tetramer, wherein two antiparallel ROCK dimers likely associate along their coiled-coil axes. This arrangement would permit head-to-tail interactions between the kinase and PH/RBD domains across opposing dimers. We speculate that this higher-order tetramerization stabilizes localized signaling hubs, enhancing avidity toward membrane-associated targets. Consequently, a regulated tetramer-to-dimer transition could serve as a elegant mechanistic switch to amplify ROCK kinase activity at discrete subcellular sites, restricting autoinhibitory head-to-tail interactions specifically to the tetrameric state.

The elongated coiled-coil region has also been proposed to act as a molecular ruler spanning 90–100 nm, which positions the kinase domain at a precise distance optimized for Myosin II phosphorylation within the crowded cell cortex^8,31^. However, this spatial separation raises a central mechanistic question: How does membrane-anchored RhoA, the canonical upstream activator of ROCK, engage with and regulate such a distant target? Recent work by Gierse et al. demonstrated that active RhoA binds directly to the C-terminal PH-C1 tandem, recruiting ROCK^FL^ to membrane-proximal activation zones^32^. While this interaction identifies PH-C1 as a RhoA-responsive docking site, it unexpectedly dampens Myosin II activation. Intriguingly, in vitro assays demonstrate that purified ROCK^FL^ exhibits robust basal kinase activity towards MYPT1, which is markedly higher than that of the isolated kinase domain—underscoring the requirement of additional structural domains for full enzymatic function. While inhibition by Y-27634 confirms the specificity of this catalytic activity, the lack of further stimulation by RhoA•GppNHp indicates that purified ROCK^FL^ already adopts a catalytically competent conformation under these cell-free assay conditions. This apparent paradox is resolved by SAXS and biochemical analyses of ROCK2, which reveal that RhoA binding does not directly enhance catalytic turnover. Instead, it redistributes the conformational ensemble towards more extended states, as evidenced by increased radius of gyration (*R*_g_) values^61^. Consequently, RhoA primarily regulates the spatial organization of ROCK, facilitating the kinase domain’s access to membrane-distal substrates rather than accelerating catalysis—thereby coupling conformational plasticity to selective signal transmission^61^. Together, these findings demonstrate that membrane recruitment and an unmasked kinase domain are insufficient for functional ROCK activation; rather, precise long-range conformational transitions and cooperative domain interactions are required to orchestrate downstream signaling.

Our observation that ROCK^FL^ exhibits robust baseline kinase activity under cell-free conditions—uncoupled from further enhancement by active RhoA—yields critical insights into its basal state. Combined with our structural reconstitutions, these data strongly suggest that the fully extended, parallel conformation represents the default baseline state of the isolated protein. This diverges significantly from classical activation models that postulate an inherent, high-affinity intramolecular autoinhibitory fold in solution. Consequently, maintaining a autoinhibited state within the cellular environment must depend on external cellular factors absent in our assays. In cells, the spatial constraints of the cortical actin meshwork, macromolecular crowding, or specific anchoring sites at the plasma membrane may be mandatory to mechanically restrict the kinase domains. RhoA engagement at the C-terminal PH-C1 tandem domain may therefore function as a spatial targeting, indexing, and orientation mechanism rather than a disruptive conformational switch.

This striking 100 nm molecular reach, corroborated by our negative-stain EM analysis, is physiologically essential for downstream signal transduction. By acting as an extended molecular scaffold, ROCK1 effectively bridges distinct spatial microenvironments. While the C-terminal RBD and PH-C1 domains anchor the enzyme to active RhoA and phosphoinositide pools at the plasma membrane, the parallel coiled-coil projects the N-terminal catalytic domains deep into the subcortical cytoplasm. This broad physical reach allows the kinase domain to access and phosphorylate macromolecular cytoskeletal targets, such as MYPT1 and the myosin light chain, which reside within dense actomyosin networks that are sterically inaccessible to short, globular or strictly membrane-bound signaling enzymes. The oligomeric plasticity observed across different fragments suggests that the central coiled-coil region serves as a functional switch modulating effector activity (Table 1 and Fig. 3A). Rho effectors are remarkably diverse; for instance, unlike ROCK, PKN1 operates as a monomeric protein kinase under physiological conditions.

Our AUC data suggest that partial unfolding of PKN1^HR1^ yields a compact, flexible conformation that sediments rapidly relative to its mass. Conversely, the dimeric ROCK^HR1^ and tetrameric RTKN^HR1^ coiled-coil bundles adopt highly elongated rod-like structures with large frictional coefficients, which retards their sedimentation disproportionately to their respective molecular masses of 31 kDa and ~40 kDa, respectively (Table 1 and Fig. 2). Interestingly, RTKN^HR1^ differs significantly from PKN1^HR1^ despite sharing 40% sequence, with our data indicating that RTKN^HR1^ adopts a distinct α-helical tetrameric structure, though its precise physiological role remains to be elucidated.

A critical structural feature resolved here is the heptad periodicity and coiled-coil forming potential of ROCK^FCC^. The structural stutter identified at the C-terminus of ROCK^HR1^ indicates a disruption in the heptad repeats, resulting in a non-canonical right-handed coiled coil pitch. Extending the sequence following L547 in ROCK^HR1^ fails to align with the regular heptad repeats in the ROCK^CC^ structure^26^, strongly indicating the presence of a second coiled-coil discontinuity, such as a skip, stammer, or stutter^53^. This is reminiscent of ROCK^SBD^, which features a C-terminal helical bundle where disrupted heptad repeats force adjacent helices into an antiparallel orientation^18^. Rather than acting as a rigid molecular ruler, these periodic discontinuities introduce sites of localized mechanical compliance. These regions act as molecular hinges or universal joints, permitting torsional bending and rotation along the long axis of the coiled-coil. This conformational freedom is likely essential to allow the flanking kinase domains to navigate and access bulky substrates within the crowded cortical environment. Ultimately, obtaining a high-resolution structure of ROCK^FL^ will clarify whether these discontinuities define natural, functionally discrete mechanosensitive subdomains in the core engine.

### Conclusions and future perspectives

In summary, our biochemical, biophysical, and structural data reveal that ROCK^HR1^ forms a parallel coiled-coil dimer. This structure is distinct from the antiparallel PKN^HR1^ and the tetrameric RTKN^HR1^ assembly. High-resolution X-ray structural data, supported by SAXS, reveal that ROCK^HR1^ forms an elongated, rod-shaped coiled-coil. This coiled-coil is characterized by canonical heptad packing and a localized stutter that imparts torsional flexibility. EM data combined with molecular modeling of the ROCK^FL^ protein further establishes this extended dimeric architecture, providing a structural rationale for conformational modulation during downstream signaling. This extended framework aligns with the molecular-ruler model originally proposed by Truebestein et al.^30^ and recently refined by Gierse et al.^32^. Together, these insights indicate that the full-length architecture of ROCK actively coordinates spatial regulation rather than serving as a passive structural spacer. In concert with the PH-C1 tandem, the elongated coiled-coil orchestrates membrane recruitment, precise positioning of the catalytic domain, and localized Myosin II activation—thereby ensuring that actomyosin contractility remains finely tuned to specific cellular contexts.

Mechanistically, these findings redefine our understanding of how the dynamic ROCK dimer transduces RhoA-mediated signals. By coupling structural rigidity with mechanical plasticity, the coiled-coil core works cooperatively with the RhoA-binding domain (RBD) and the C-terminal PH-C1 domains. Together, they communicate membrane-proximal activation events directly to the distant kinase domain.

Looking forward, reconstituting purified ROCKFL into nanodiscs or compositionally tailored liposomes will be instrumental in capturing its membrane-bound conformational states via high-resolution cryo-EM. These biomimetic approaches are essential for determining how membrane engagement, Rho binding, and long-range coiled-coil transitions are structurally coupled. Varying the lipid composition systematically will allow us to distinguish between specific membrane insertion and non-specific electrostatic association. This will help us define lipid-dependent regulatory modes that may modulate ROCK activation. Extending these reconstitution strategies to time-resolved and in situ approaches will be critical for capturing the transient dynamics of ROCK during active Rho signaling and mechanical actomyosin remodeling.

Given the central role of ROCK signaling in myosin-driven contractility, centrosome-associated processes, cancer cell invasion, fibrosis, and cardiovascular disease, elucidating these membrane-associated conformational transitions is critical for understanding disease-associated dysregulation^62–64^. Ultimately, deciphering this long-range regulatory mechanism paves the way for structure-guided therapeutic strategies that can selectively modulate ROCK function. This moves the field beyond non-specific, global kinase inhibition and toward highly precise, conformation-targeted interventions.

## Supplementary information

Transparent Peer Review File

Supplementary Information

## Data Availability

The atomic coordinates and structure factors for dimeric, parallel ROCK^HR1^ have been deposited in the Protein Data Bank (PDB) under the ID 8P0S.
