# Supplementary material for: Structural and functional insights into the p160 Rho-associated coiled-coil-containing protein kinase ROCK
Source: Commun Chem. 2026 Nov 4;9:344. doi: 10.1038/s42004-026-02258-2 (PMC13737125; doi:10.1038/s42004-026-02258-2)
Supplement: Supplementary file 1 [file 42004_2026_2258_MOESM1_ESM.pdf]

nature portfolio

Peer Review File

Structural and functional insights into the p160 Rho-associated coiled-coil-containing protein kinase ROCK

Corresponding Author: Dr Badri Dubey

This file contains all reviewer reports in order by version, followed by all author rebuttals in order by version.

Version 0:

Reviewer comments:

Reviewer #1

(Remarks to the Author)
This manuscript describes an interesting study on ROCK1, a kinase that is a downstream effector for RhoA, and an important disease target. The authors have used a number of biophysical and structural techniques to determine how ROCK1 forms homodimers and have used the existing structural information along with the structure they have solved to put forward a model for full-length ROCK1. Overall this is an interesting study of interest to a wide audience that is suitable for publication subject to some revision as detailed below.

Abstract – using the abbreviation ROCK^FCC but this is not standard so should be removed. Also the ROCK^HR1 which only appears once in the abstract.
Line 93 Gierse et al should be numbered reference (currently 32)

Figure 1
It would make more sense to switch panel A and panel B. Then the ROCK constructs are shown first, which is the focus of this paper. Also, it would mean that panel C would be in the right place.

Figures 1A/B – it is not clear what homology is being represented by the shading. Is this homology across a larger number of proteins, or across those shown? Also why are D and E not considered to have similar properties when R and K are?

In the text (line 272) it is stated that the ROCK-type HR1 does not share homology with the PKN-type HR1 but they are shown in separate alignments, which makes it hard to judge. It would be helpful if they were also shown in a single sequence alignment (e.g. in the supplementary material) to make it clear.

Figure S1
The CD suggests that PKN is not fully folded – it has a significantly different shape to the other two spectra. When something similar was observed for ROCK RBD in Supplementary S4 it was suggested (line 421) that this is due to partial unfolding. The same explanation applies here. PKN also has an anomalous mass in SEC-MALS experiments: the ROCK and Rhotekin HR1 domains shown are close to the expected mass for a dimer and tetramer respectively. Both are slightly lower, consistent with the idea that there is some monomer present. PKN is however much lower than the expected mass and is a monomer. There are no values given in the text for the uncertainty in these numbers and this should be added.

In the methods it is not stated what concentration the proteins were in the SEC-MALS. Instead, there is an explanation about how MALS works and why it is useful. This should be removed but the concentrations of the proteins used in the experiments should be added. This is required because the species observed in MALS and AUC are different.

The masses estimated from the AUC data should be included in Table 1. All species detected in the AUC should be labelled in Figure S1C alongside their calculated masses. Finally, there should be some explanation as to why the PKN1 monomer (12 kDa) has a similar S value to the ROCK dimer (31 kDa) and its dimer (24 kDa) is similar to the RTKN tetramer (40 kDa).

Figure S2 SAXS: the end of the legend states that data are summarized in Table 1, but there is nothing from the SAXS in Table 1. Please add the SAXS summary to Table 1, or correct the figure legend. Given that the Dmax are quoted please

could the authors include the P(r) plots.

Figure 2C: the E442 and K446 contacts with their counterpart on the other helix are suggested to be responsible for the increased radius of the coiled-coil between 440 and 450. However, in Figure 2C it seems that E442 is not contacting the same residue on the opposite chain, but is instead contacting a Met which is not identified in that panel. Please can this be clarified, and all interacting residues labelled or the orientations changed to make it more obvious? I also suggest that these very detailed panels Figure 2B-E would be better placed in the Supplementary data, and that the CD, SEC-MALS and AUC data for ROCK are moved into the main figures, alongside the structure of the ROCK dimer.

Figure 3 represents an important attempt to model the structure of full-length ROCK, based on the individual domains and negative stain EM. It is very unclear how this modelling was carried out however. The text suggests (line 404-405) that it is detailed in the methods, but I cannot find this information there. Given the interesting results obtained please can more details be added to the manuscript.

Line 466: “full length ROCK showed the best association with Folch-I liposomes”. Since the authors went to the effort of describing their composition in the methods, please can the sedimentation assays for the other types of liposomes be included in the Supplementary figures.

Assuming that the PH/C1 domains are mediating the liposome interactions, do the negative stain results with liposomes help to resolve which globular entity observed in Figure 3C is the kinase?

Lines 481-483: the authors find that FL ROCK has a higher activity than the kinase alone and state that “The kinase domain contains short N- and C-terminal extensions that have been reported to facilitate head-to-head dimerization of the kinase lobes and thus full functionality”. It is not clear from this whether they are putting forward an explanation for the lower activity – is that the case? If it is, why were the short extensions not included in the kinase constructs that were used for the expression?

In the Discussion (line 516) the dimerization of ROCK was suggested to allow interactions between the head and tail domains. The model shown in Figure 3A would not support this – could the authors please clarify?

Reviewer #2

(Remarks to the Author)
Dubey et al. presents an in-depth structural study of the central coiled-coil region of human ROCK1. Using SAXS, analytical ultracentrifugation, negative-stain EM, SEC-MALS, and X-ray crystallography, they characterize fragments spanning the HR1 (RhoA-binding) domain through the entire 680–700-residue coiled-coil (ROCKFCC). All these studies, coupled with the 2.2 Å crystal structure of the ROCK1HR1 domain, show a parallel two-stranded coiled-coil dimer. Furthermore, sequence analysis reveals two distinct classes of Rho effector HR1 domains (“ROCK-type” vs. “PKN-type”) with different lengths and motifs, and EM data indicate that the full ROCK1 coiled-coil forms a highly elongated (~100–120 nm) parallel dimer. Notably, EM of the isolated coiled coil (ROCKFCC) shows it can assemble into tetrameric bundles in addition to dimers. A pseudo-atomic model of full-length ROCK1 (combining known kinase and PH-C1 structures with the coiled-coil) places the N-terminal kinase dimer and C-terminal PH-C1-PH domains more than 100 nm apart, and functional assays show that purified full-length ROCK1 is highly active on substrate (MYPT1) and is not further stimulated by GTP-bound RhoA, consistent with a constitutively “extended” active state. Taken together, this exhaustive characterization proposes that ROCK forms a rigid, rod-like dimer that likely acts as a “molecular ruler” to position its kinase domains relative to membranes, rather than being regulated by autoinhibition.
The major strength of this study is that the authors provide the first experimental evidence that the ROCK1HR1 domain forms a parallel coiled coil dimer, which corrects previous models suggesting an antiparallel arrangement.
However, the study has some drawbacks:
1. The authors observe that ROCKFL is constitutively active and not further stimulated by RhoA. They should clarify if this suggests that the "extended" form is the canonical state and whether cellular factors (missing in vitro) are required to maintain autoinhibition. Expanding the discussion on how the 100 nm distance affects signal transduction is critical.
2. Both ROCKFCC and RTKNHR1 show a tendency toward tetramerization. The authors need to address whether this is a physiological state or potentially an artifact of high concentrations in biophysical assays. What is the biological significance of this tetramerization is something that the authors should shed light on.
3. The manuscript mentions a "stutter" in the heptad repeat of ROCKHR1 and other potential discontinuities in the full coiled coil. The authors should discuss whether these sites act as flexible hinges rather than rigid ruler segments, allowing the kinase domain to reach varied substrates.
4. Since HR1 is a known RhoA binding motif in other kinases, it would be valuable to test whether ROCK1’s HR1 binds RhoA-GTP, and if so, how? The authors measure RhoA’s effect on activity but does not test direct binding to the new HR1 domain. If RhoA fails to bind HR1, that fact itself would be important to report. Some biophysical studies like FRET or SPR to validate would be meaningful.
5. From Figure 3D, how do the authors interpret the specificity of the domain of ROC1 interacting with liposome? A quantification of how many ROC1s are engaged to liposome vs free liposome will be useful. Furthermore, correlating that to the pelleting assay can further strengthen this observation.
Overall, this is a decent manuscript, where the authors should address the points raised above before it can be accepted.

Reviewer #3

(Remarks to the Author)
I co-reviewed this manuscript with one of the reviewers who provided the listed reports. This is part of the Communications Chemistry initiative to facilitate training in peer review and to provide appropriate recognition for Early Career Researchers who co-review manuscripts.

Version 1:

Reviewer comments:

Reviewer #1

(Remarks to the Author)
The manuscript is much improved and almost ready for publication subject to minor corrections. Although I was not provided with a tracked version of the manuscript to be able to see what parts had changed, most of my comments have been addressed. I accept the authors argument about any changes that I requested that have not been made.

Two issues remain:
1. My understanding is that SEC-MALS gives an absolute mass based on the refractive index, independent of the shape or folding of the protein. In that sense it is less reliant on fitting to a model than AUC. In the added text (line 341) it is suggested that the unfolded nature of PKN1 HR1 means that the MALS-based molecular mass is incorrect because of partial unfolding. Can the authors please provide a reference for this statement given that it contradicts the accepted view about MALS? In Figure 2B the shape of the mass line for this protein is very different to that for the masses of the other two proteins shown in the same panel. The mass lines for ROCK and RTKN are humped, whereas that for PKN1 is sloping downwards from left to right, implying that the peak is a mixture of species and that the mass cannot be accurately determined.
2. I asked for some explanation for the S values and this was provided at length in the rebuttal letter from the authors. Sorry that this was unclear, I was requesting an explanation in the manuscript. Please can a brief note go into the text? It does not have to be as extensive as the explanation provided, just a simple sentence will be sufficient.

Reviewer #2

(Remarks to the Author)
The authors have satisfactorily addressed all the concerns raised and have substantially improved the manuscript. We therefore recommend that the manuscript be accepted for publication without further delay.

Reviewer #3

(Remarks to the Author)
I co-reviewed this manuscript with one of the reviewers who provided the listed reports. This is part of the Communications Chemistry initiative to facilitate training in peer review and to provide appropriate recognition for Early Career Researchers who co-review manuscripts.

Open Access This Peer Review File is licensed under a Creative Commons Attribution 4.0 International License, which permits use, sharing, adaptation, distribution and reproduction in any medium or format, as long as you give appropriate credit to the original author(s) and the source, provide a link to the Creative Commons license, and indicate if changes were made.
In cases where reviewers are anonymous, credit should be given to 'Anonymous Referee' and the source.
The images or other third party material in this Peer Review File are included in the article's Creative Commons license, unless indicated otherwise in a credit line to the material. If material is not included in the article's Creative Commons license and your intended use is not permitted by statutory regulation or exceeds the permitted use, you will need to obtain permission directly from the copyright holder.
To view a copy of this license, visit https://creativecommons.org/licenses/by/4.0/

Responses to the reviewers' comments on the manuscript COMMSCHEM-26-0138-T:

Reviewer #1
This manuscript describes an interesting study on ROCK1, a kinase that is a downstream effector for RhoA, and an important disease target. The authors have used a number of biophysical and structural techniques to determine how ROCK1 forms homodimers and have used the existing structural information along with the structure they have solved to put forward a model for full-length ROCK1. Overall this is an interesting study of interest to a wide audience that is suitable for publication subject to some revision as detailed below.

Author's response: We would like to thank the reviewer for the positive and encouraging assessment of our study on ROCK1. We appreciate the recognition of our work's relevance to a wide audience and the value of our biophysical and structural approach in modeling the full-length protein. We have carefully addressed the suggested revisions, which has further strengthened the manuscript and clarified our proposed model of the ROCK1 homodimer.

Abstract – using the abbreviation ROCK^FCC but this is not standard so should be removed. Also the ROCK^HR1 which only appears once in the abstract.

Author's response: Thank you for this helpful suggestion. We have revised the abstract to improve clarity and adhere to standard nomenclature. Specifically, we have replaced the non-standard abbreviation ROCK^FCC with the full term 'full coiled-coil domain of ROCK'. Additionally, we have replaced the single occurrence of ROCK^HR1 with 'homology region 1 of ROCK'.

Line 93 Gierse et al should be numbered reference (currently 32)

Author's response: We thank the reviewer for highlighting this issue. It has now been corrected and numbered 32 in the revised manuscript.

Figure 1
It would make more sense to switch panel A and panel B. Then the ROCK constructs are shown first, which is the focus of this paper. Also, it would mean that panel C would be in the right place.

Author's response: While we appreciate the reviewer's suggestion to reorder the panels to highlight the ROCK constructs earlier, we have decided to maintain the current organization. The current sequence of panels A, B, and C directly mirrors the logical flow of the Results section. Changing the order would require a significant restructuring of the text and, in our view, would disrupt the step-by-step narrative we have established to guide the reader through the story.

Figures 1A/B
It is not clear what homology is being represented by the shading. Is this homology across a larger number of proteins, or across those shown?

Author's response: Regarding Figures 1A/B, we clarify that the shading represents homology across only the specific proteins shown in the alignment. We have updated

the figure legend to explicitly state the criteria used for shading (e.g., identity vs. similarity) and the reference group.

Also why are D and E not considered to have similar properties when R and K are?

Author's response: We appreciate the reviewer's astute observation regarding the biochemical properties of these residues. You are absolutely right that E/D should be treated with a similar logic as R/K. Accordingly, we have revised the alignments in Figures 1A and 1B to consistently reflect the homology for both acidic and basic residues. The shading has been updated to ensure scientific consistency throughout the figure.

In the text (line 272) it is stated that the ROCK-type HR1 does not share homology with the PKN-type HR1 but they are shown in separate alignments, which makes it hard to judge. It would be helpful if they were also shown in a single sequence alignment (e.g. in the supplementary material) to make it clear.

Author's response: We have carefully considered the suggestion to align the HR1 domains of ROCK and PKN. However, we have decided not to include this comparison for the following structural reasons: (1) Size Discrepancy: The HR1 domains of ROCK and PKN differ significantly in length, with one being nearly double the size of the other. (2) Alignment Artifacts: Standard alignment algorithms would force a 'stretching' of the sequences to find a match. While this might yield some mathematical homology due to the shared alpha-helical nature of these domains, such an alignment would likely be structurally misleading and lack biological relevance. (3) Structural Basis: Since both domains are predominantly alpha-helical, a sequence-based alignment might show artificial similarities that do not reflect true evolutionary or functional conservation. Given these constraints, we believe that forcing an alignment would decrease the clarity and accuracy of the figure rather than adding valuable insight.

Figure S1
The CD suggests that PKN is not fully folded – it has a significantly different shape to the other two spectra. When something similar was observed for ROCK RBD in Supplementary S4 it was suggested (line 421) that this is due to partial unfolding. The same explanation applies here. PKN also has an anomalous mass in SEC-MALS experiments: the ROCK and Rhotekin HR1 domains shown are close to the expected mass for a dimer and tetramer respectively. Both are slightly lower, consistent with the idea that there is some monomer present. PKN is however much lower than the expected mass and is a monomer. There are no values given in the text for the uncertainty in these numbers and this should be added.

Author's response: We thank the reviewer for this insightful observation. We agree that the CD spectrum of $PKN1^{HR1}$ differs significantly from $ROCK^{HR1}$ and $RTKN^{HR1}$. We have revised the manuscript to state that this anomalous spectral profile indicates a partially unfolded state, which is consistent with our interpretation of the $ROCK^{RBD}$ in Supplementary Figure S4. We have also expanded the discussion of the anomalous SEC-MALS behavior of $PKN1^{HR1}$, including values for all reported molecular masses in Table 1.

The revised manuscript now includes the following text on line 345:

The CD spectrum of PKN1^{HR1} (1–106) exhibits a distinct profile. Notably, there are fewer minima at 220 nm than in the spectra of ROCK^{HR1} and RTKN^{HR1}. This suggests a partially unfolded conformational state. This is consistent with SEC-MALS analysis yielding a molecular mass of 8.9 kDa, which is substantially lower than the theoretical monomeric mass of 11.8 kDa. This indicates the presence of a monomeric species that is likely to be partially unfolded in solution (Figure S1 and Table 1). AUC analysis revealed a heterogeneous population comprising 46% monomer (11.2 kDa) and 51% dimer (22.4 kDa). Although PKN1^{HR1} predominantly behaves as an undersized monomer in SEC-MALS experiments, these results suggest that it retains a limited propensity for self-association under sedimentation conditions. The lower apparent molecular mass measured by MALS is consistent with partial unfolding, which may alter hydrodynamic properties and reduce refractive index contributions. Taken together, the SEC-MALS, AUC and CD data provide convergent evidence that PKN1^{HR1} does not adopt a stable, fully folded coiled-coil conformation under the tested experimental conditions. This contrasts with the well-ordered dimeric and tetrameric assemblies observed for ROCK^{HR1} and RTKN^{HR1}, respectively.

In the methods it is not stated what concentration the proteins were in the SEC-MALS. Instead, there is an explanation about how MALS works and why it is useful. This should be removed but the concentrations of the proteins used in the experiments should be added. This is required because the species observed in MALS and AUC are different.

Author's response: We thank the reviewer for this correction. As suggested, we have removed the general explanation of the MALS methodology from the 'Methods' section to make it more concise. As requested, we have included the specific protein concentrations used for these experiments.

For the SEC-MALS runs, the proteins were injected at a concentration of 0.5 mg/ml in a volume of 100 µl. We have updated the Methods section accordingly.

The masses estimated from the AUC data should be included in Table 1. All species detected in the   should be labelled in Figure S1C alongside their calculated masses.

Author's response: We thank the reviewer for these constructive suggestions. In Table 1, we present the estimated molecular masses derived from AUC alongside the MALS values for direct comparison. Table 1 also includes the masses derived from AUC, alongside the respective population percentages for each species detected in the c(s) distribution. As requested, Figure S1C which is Fig. 2B has been updated to label all species detected in the AUC c(s) distribution, along with their assignments (monomer, dimer, or tetramer) and calculated molecular masses.

Finally, there should be some explanation as to why the PKN1 monomer (12 kDa) has a similar S value to the ROCK dimer (31 kDa) and its dimer (24 kDa) is similar to the RTKN tetramer (40 kDa).

Author's response: We thank the reviewer for highlighting this important point. The sedimentation coefficient (S) depends on the molecular mass, shape and hydrodynamic radius of the sedimenting particle. This is described by the Svedberg equation: $S = M(1 - \bar{v}\rho) / Nf$, where Nf is the sedimentation velocity, f is the frictional coefficient and M is the molecular mass. Particles that are more elongated or extended

particles have larger frictional coefficients and therefore sediment more slowly than compact, globular particles of the same mass. This explains why the PKN1^{HR1} monomer (with a mass of ~12kDa) and the ROCK^{HR1} dimer (with a mass of ~31kDa) sediment at similar *S*-values, as do the PKN1^{HR1} dimer (with a mass of ~24kDa) and the ROCK^{HR1} tetramer (with a mass of ~40kDa). ROCK^{HR1} forms a well-ordered, parallel coiled-coil dimer, which is a highly elongated, rod-like structure with a large frictional coefficient relative to its mass. This extended shape retards sedimentation, causing the 31kDa dimer to sediment at a comparable *S*-value to the smaller, more compact (or partially unfolded) PKN1^{HR1} monomer. Similarly, RTKN^{HR1} assembles into a tetrameric coiled-coil bundle. Despite its larger mass (approximately 40 kDa), it is also an elongated assembly with a correspondingly high frictional coefficient. This causes it to sediment at a similar *S*-value to the PKN1^{HR1} dimer (approximately 24kDa). PKN1^{HR1} is likely to be more compact and flexible due to its partial unfolding. This is consistent with its anomalous CD spectrum and sub-monomer MALS mass. Therefore, it sediments faster relative to its mass compared to the well-folded coiled-coil assemblies of ROCK^{HR1} and RTKN^{HR1}.

Figure S2 SAXS: the end of the legend states that data are summarized in Table 1, but there is nothing from the SAXS in Table 1. Please add the SAXS summary to Table 1, or correct the figure legend. Given that the Dmax are quoted please could the authors include the P(r) plots.

Author's response: We thank the reviewer for this valuable suggestion. We agree that the statement in the Figure S2 legend indicating that the SAXS data are summarized in Table 1 was missing, as the SAXS parameters were not included in the table. To address this issue, we have now included the SAXS summary parameters in Table 2 and have added the P(r) plots to the revised **supplementary figures S4D** as panel.

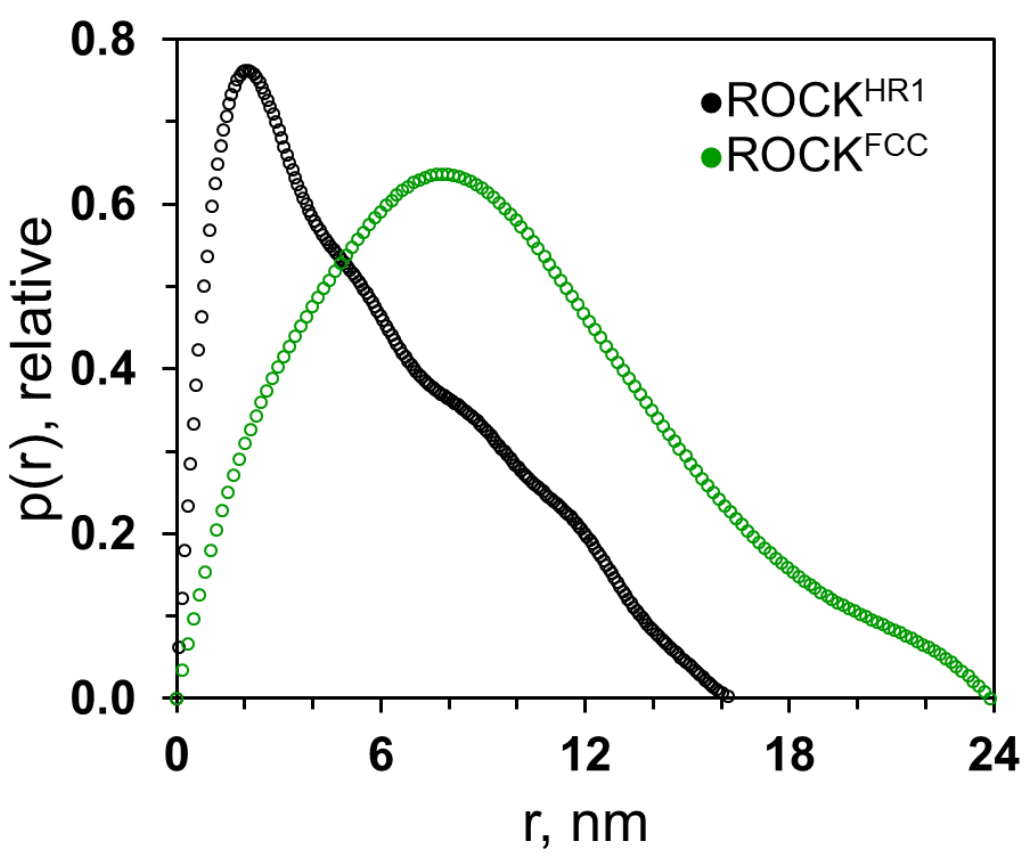

Figure S2. (D) P(r) plots plots of ROCK^{HR1} and ROCK^{FCC}. All data are summarized in Table 2.

Table 2. SAXS data collection and analysis of ROCK^{HR1} and ROCK^{FCC}

**Data collection**	ROCK^{HR1}	ROCK^{FCC}
**Data collection**		
Radiation source	X33 (DORIS @ DESY, Germany)	
s-range (nm^{-1})	0.08 – 6.0	
Exposure time (s)	15	
**Structural Parameters**		
R_G (from Guinier, Å)	41 ± 1.1	78 ± 3.3

R_G (from Guinier, Å)	48 ± 0.1	73 ± 4.0
D_{max} (Å)	165	239
**Software Utilized**		
Data reduction & averaging	PRIMUS	
Guinier and P(r) distribution	GNOM	
*M*odelling programs	Dammif, Crysol	

Figure 2C: the E442 and K446 contacts with their counterpart on the other helix are suggested to be responsible for the increased radius of the coiled-coil between 440 and 450. However, in Figure 2C it seems that E442 is not contacting the same residue on the opposite chain, but is instead contacting a Met which is not identified in that panel. Please can this be clarified, and all interacting residues labelled or the orientations changed to make it more obvious?

Author's response: We thank the reviewer for this careful observation. Using TWISTER, we identified seventeen heptad repeats in ROCK ^{HR1} (Q425–L548). Fig. 2 Panel B (revised Fig. S1 B in current manuscript) shows the 'knobs-into-holes' canonical contact, where the A and D positions are occupied by typical hydrophobic residues (Leu, Ile, Val and Ala). Panels C–E illustrate the structural consequences of having non-hydrophobic residues in these positions. Rather than destabilizing the coiled coil, the structure adapts locally: the side chains reorient and substitute the sterically or electrostatically incompatible canonical contacts with neighboring residues. A four-residue insertion (SQNS, positions 530–533; stutter) marks the transition from left-handed to right-handed geometry. This explains why five of the six polar interactions in panel E cluster in the post-stutter region, where the fold is no longer canonical.
The reviewer is correct that E442 does not interact with the corresponding residue on the other chain. This is intentional and reflects the ROCK coiled-coil dimer described in lines 420–426. E442 and K446 are atypical heptad-position residues that cannot be packed ergonomically within the coiled coil using the traditional knobs-into-holes method. Since placing two equally charged residues (e.g., Glu/Glu or Lys/Lys) in equivalent a/d positions on opposing helices generates electrostatic repulsion, the local backbone geometry adjusts. The helices splay slightly, the charged side chains reorient to direct their carboxylate or amino groups away from the interface, and a neighboring residue comes into contact to mediate the interaction. For E442 specifically, the neighboring partner is M449 on the opposite chain. It forms a polar–apolar interaction with M449. This contact is depicted in panel 2C. M449 was visible in the panel but unlabeled, which caused confusion.

I also suggest that these very detailed panels Figure 2B-E would be better placed in the Supplementary data, and that the CD, SEC-MALS and AUC data for ROCK are moved into the main figures, alongside the structure of the ROCK dimer.

Author's response: We agree with the reviewer's suggestion regarding the figure organization. Moving the biophysical characterization (CD, SEC-MALS, and AUC) into the main figures alongside the ROCK dimer structure significantly strengthens the narrative and provides a more comprehensive overview of our key findings. Accordingly, we have moved the detailed panels from Figure 2 to the Supplementary Data (now Supplementary Figure S1). The main figures have been restructured to

integrate the ROCK biophysical data as suggested. We believe this new arrangement greatly improves the clarity and impact of the manuscript.

Figure 3 represents an important attempt to model the structure of full-length ROCK, based on the individual domains and negative stain EM. It is very unclear how this modelling was carried out however. The text suggests (line 404-405) that it is detailed in the methods, but I cannot find this information there. Given the interesting results obtained please can more details be added to the manuscript.

Author's response: We thank the reviewer for pointing out this important issue. We agree that the methodology behind the composite model in Figure 3A was not explained in the manuscript. This model is essential to our structural interpretation of the architecture of full-length ROCK. In response, we added a new Methods subsection titled "Composite Structural Model of Full-Length ROCK," detailing the following steps: (i) the experimentally determined PDB structures used for each ROCK domain; (ii) modeling interdomain linkers and regions without experimental structural information using the Dolly server; (iii) assembling the experimentally determined and predicted structures into a single composite model in COOT by aligning the overlapping terminal residues; and (iv) performing structural visualization and dimensional analysis in PyMOL. We believe this expanded description provides sufficient transparency and reproducibility, thereby addressing the reviewer's concern. The following part was added to the Methods section in line 278:

Composite structural model of full-length ROCK
A composite structural model of full-length ROCK was generated by integrating experimentally determined domain structures with predicted models for regions lacking structural information. The final model encompassed the entire ROCK polypeptide, extending from the N-terminal kinase domain to the C-terminal pleckstrin homology (PH) domain. Experimentally determined structures were obtained from the Protein Data Bank and used as template structures to assemble a grand final template for homology modelling. These experimental structures in included the kinase domain dimer (PDB 2ESM), ROCKHR1 (PDB 8P0S; this study), ROCKCC (PDB 3O0Z), ROCKSBD (PDB 2L4W), ROCKRBD (PDBs 1S1C and 1UIX), and the PHn–C1–PHc region (PDB 2ROV). All structures were used as and crystallographic symmetry mates, solvent molecules, and non-essential ligands were removed Prior to assembly. First, a canonical coiled-coil structure was generated for the central region of ROCK (residues 423–1011), which is predicted to adopt this fold. Second, all known structures for this region were superposed onto their corresponding parts. Fragments of the canonical core that overlapped with the template structures were then removed. For overlapping template structures, regions closer to the canonical fold were retained while the remaining parts were removed. Additionally, residues from structures that deviated by more than 3.0 Å from the canonical core were excluded. In the third step, the kinase domain, as well as the PH and C1 domains, were manually placed at the N- and C-termini of the coiled-coil template, respectively, using PyMOL. They were placed and oriented in plausible positions to leave sufficient space for the missing loops, thereby creating the complete template. Finally, after renumbering all residues in the template in accordance with the full-length sequence of ROCK, MODELLER was used to generate a homology model of the full-length protein.
The resulting full-length ROCK composite model spanned approximately 100 nm, consistent with dimensions observed by negative-stain electron microscopy. Structural visualization and distance measurements were performed in PyMOL. The overall

architecture of the composite model was assessed qualitatively by comparison with negative-stain EM class averages, with which it showed good agreement.

Line 466: "full length ROCK showed the best association with Folch-I liposomes". Since the authors went to the effort of describing their composition in the methods, please can the sedimentation assays for the other types of liposomes be included in the Supplementary figures.

Author's response: We thank the reviewer for this point. While we indeed tested various liposome compositions during our initial screening, the sedimentation assays for compositions other than Folch-I were part of a preliminary optimization phase. Unfortunately, these early data sets were not recorded to a publication-quality standard and were therefore not archived for formal presentation. However, these preliminary runs consistently indicated that Folch-I provided the most robust and reproducible binding environment for full-length ROCK. We have clarified this in the text (Lines-500-504) by referring to these observations as 'preliminary screening data'.

Assuming that the PH/C1 domains are mediating the liposome interactions, do the negative stain results with liposomes help to resolve which globular entity observed in Figure 3C is the kinase?

Author's response: We thank the reviewer for this comment. No, the negative stain results unfortunately do not help to resolve which globular entity is the kinase. This would require additional experiments, for example, using nano gold labels attached to one of the globular domain or checking the binding of truncated versions of ROCK to liposomes.

Lines 481-483: the authors find that FL ROCK has a higher activity than the kinase alone and state that "The kinase domain contains short N- and C-terminal extensions that have been reported to facilitate head-to-head dimerization of the kinase lobes and thus full functionality". It is not clear from this whether they are putting forward an explanation for the lower activity – is that the case? If it is, why were the short extensions not included in the kinase constructs that were used for the expression?

Author's response: The reviewer raises an important point regarding the construct design. Our rationale for using the truncated kinase domain was based on previously established protocols in the literature [Leung et al., 1996], which aimed to define the minimal catalytic core. At the time of expression, these short N- and C-terminal extensions were excluded to maximize protein stability and solubility during purification, as longer constructs in our hands showed a higher tendency for aggregation. It remains unclear whether the absence of these extensions is likely to explain the lower activity observed compared to that of the full-length (FL) protein.

We have updated the manuscript to explicitly state (Lines 522-523) that the reduced activity of the kinase-only construct is likely a direct consequence of lacking these regulatory extensions, rather than an inherent property of the catalytic mechanism itself.

In the Discussion (line 516) the dimerization of ROCK was suggested to allow interactions between the head and tail domains. The model shown in Figure 3A would not support this – could the authors please clarify?

Author's response: We thank the reviewer for the careful reading of our manuscript and for identifying this inconsistency. We apologize for the confusion.
We have added a paragraph addressing this point in lines 565–575.

We observed that ROCK^{FCC} transitions into higher-order oligomers at micromolar concentrations. In the crowded cellular environment, local ROCK concentrations can increase dramatically via macromolecular crowding or scaffold-mediated membrane recruitment. Our AUC data demonstrate that a discrete fraction of ROCK^{FCC} sediments as a defined tetramer, wherein two antiparallel ROCK dimers likely associate along their coiled-coil axes. This arrangement would permit head-to-tail interactions between the kinase and PH/RBD domains across opposing dimers. We speculate that this higher-order tetramerization stabilizes localized signaling hubs, enhancing avidity toward membrane-associated targets. Consequently, a regulated tetramer-to-dimer transition could serve as an elegant mechanistic switch to amplify ROCK kinase activity at discrete subcellular sites restricting autoinhibitory head-to-tail interactions specifically to the tetrameric state.

Reviewer #2
Dubey et al. presents an in-depth structural study of the central coiled-coil region of human ROCK1. Using SAXS, analytical ultracentrifugation, negative-stain EM, SEC-MALS, and X-ray crystallography, they characterize fragments spanning the HR1 (RhoA-binding) domain through the entire 680–700-residue coiled-coil (ROCKFCC). All these studies, coupled with the 2.2 Å crystal structure of the ROCK1HR1 domain, show a parallel two-stranded coiled-coil dimer. Furthermore, sequence analysis reveals two distinct classes of Rho effector HR1 domains ("ROCK-type" vs. "PKN-type") with different lengths and motifs, and EM data indicate that the full ROCK1 coiled-coil forms a highly elongated (~100–120 nm) parallel dimer. Notably, EM of the isolated coiled coil (ROCKFCC) shows it can assemble into tetrameric bundles in addition to dimers. A pseudo-atomic model of full-length ROCK1 (combining known kinase and PH-C1 structures with the coiled-coil) places the N-terminal kinase dimer and C-terminal PH-C1-PH domains more than 100 nm apart, and functional assays show that purified full-length ROCK1 is highly active on substrate (MYPT1) and is not further stimulated by GTP-bound RhoA, consistent with a constitutively "extended" active state. Taken together, this exhaustive characterization proposes that ROCK forms a rigid, rod-like dimer that likely acts as a "molecular ruler" to position its kinase domains relative to membranes, rather than being regulated by autoinhibition.
The major strength of this study is that the authors provide the first experimental evidence that the ROCK1HR1 domain forms a parallel coiled coil dimer, which corrects previous models suggesting an antiparallel arrangement.

Author's response: We would like to thank the reviewer for the exceptionally thorough and insightful summary of our work. We are particularly gratified that the reviewer recognized the significance of our findings regarding the parallel orientation of the ROCK1 HR1 domain, which clarifies a long-standing question in the field. We appreciate the acknowledgment of our 'exhaustive characterization'. The constructive comments provided have helped us to further refine the manuscript, and we have addressed all points as detailed below.

However, the study has some drawbacks:

1. The authors observe that ROCKFL is constitutively active and not further stimulated by RhoA. They should clarify if this suggests that the "extended" form is the canonical state and whether cellular factors (missing in vitro) are required to maintain autoinhibition. Expanding the discussion on how the 100 nm distance affects signal transduction is critical.

Author's response: We thank the reviewer for this insightful comment and for highlighting these crucial aspects of ROCK1 regulation and spatial biology. We fully agree that the constitutive in vitro activity of ROCK FL and its structural elongation have profound implications for its physiological activation mechanism.

We have thoroughly expanded the Discussion section to address these points directly. Below, we clarify our rationale and detail the specific revisions made to the manuscript: (1) We have added text clarifying that our integrative biophysical data (crystallography, SAXS, and negative-stain EM) strongly support a model where the default, baseline state of purified ROCK1 is an extended, parallel coiled-coil dimer. Unlike other RHO effectors (such as PKN1) that utilize antiparallel folds to facilitate compact autoinhibition, ROCK1's parallel structural framework favors an extended topology. (2) We agree that the lack of additional RhoA stimulation under the given experimental conditions suggests that maintaining a tightly autoinhibited state may require cellular elements absent in a purified system. In the revised Discussion, we postulate that inside the cell, steric constraints, macromolecular crowding, or specific interactions with membrane lipids (via the PH-C1 tandem domain) are required to restrict the orientation of the kinase domains. RhoA binding to the PH-C1 domain or the RBD does not necessarily act as an "on/off switch" via a large conformational change, but rather serves to recruit and properly orient this already competent, extended kinase to its sub-membrane cytoskeletal targets. (3) We have significantly expanded our discussion on the functional role of ROCK1's 100 nm length. Rather than being a passive tether, this massive length acts as a physical molecular scaffold. It allows the C-terminal region to anchor to RHOA and specific phosphoinositides at the plasma membrane, while the N-terminal kinase domain projects up to 100 nm into the cortical cytoplasm. This spatial reach enables ROCK1 to phosphorylate bulky, filament-associated substrates (such as MYPT1 and Myosin II) that are localized away from the immediate plane of the lipid bilayer, overcoming spatial and steric barriers that shorter kinases cannot.

Expanded Discussion on "the Extended State and Autoinhibition" (line 602-613):
Our observation that ROCKFL exhibits robust baseline kinase activity under cell-free conditions—uncoupled from further enhancement by active RhoA—yields critical insights into its basal state. Combined with our structural reconstitutions, these data strongly suggest that the fully extended, parallel conformation represents the default baseline state of the isolated protein. This diverges significantly from classical activation models that postulate an inherent, high-affinity intramolecular autoinhibitory fold in solution. Consequently, maintaining a autoinhibited state within the cellular environment must depend on external cellular factors absent in our assays. In cells, The spatial constraints of the cortical actin meshwork, macromolecular crowding, or specific anchoring sites at the plasma membrane may be mandatory to mechanically restrict the kinase domains. RhoA engagement at the C-terminal PH-C1 tandem domain may therefore function as a spatial targeting, indexing, and orientation mechanism rather than a disruptive conformational switch.

2. Both ROCKFCC and RTKNHR1 show a tendency toward tetramerization. The authors need to address whether this is a physiological state or potentially an artifact

of high concentrations in biophysical assays. What is the biological significance of this tetramerization is something that the authors should shed light on.

Author's response: The reviewer raises a critical point regarding the oligomeric state of ROCKF^{CC} and PKN^{HR1}. We have addressed this in the revised Discussion as follows:
(1) 1. Physiological state vs. Assay conditions: We acknowledge that the tetrameric species observed in SEC-MALS and AUC occur at micromolar concentrations, which are higher than typical basal levels. However, in the cellular environment, local protein concentrations can increase dramatically due to molecular crowding or scaffold-mediated recruitment to membranes. Therefore, while high concentrations in vitro favor tetramerization, similar conditions may exist in vivo under specific signaling contexts.
(2) 2. Biological Significance: We propose that the tendency toward tetramerization may represent a mechanism for higher-order clustering of ROCK or its substrates. Such clusters are often required for the formation of stable signaling hubs or for increasing the avidity of interactions with membrane-bound targets (e.g., liposomes or receptors). This switch from a dimer to a tetramer, or vice versa, could serve as a regulatory step that amplifies ROCK activity in localized subcellular sites.
To shed light on these possibilities, we have added the following paragraph to the Discussion section (line 565-575), explicitly discussing tetramerization as a potential regulatory mechanism rather than merely a technical artifact:

We observed that ROCK^{FCC} transitions into higher-order oligomers at micromolar concentrations. In the crowded cellular environment, local ROCK concentrations can increase dramatically via macromolecular crowding or scaffold-mediated membrane recruitment. Our AUC data demonstrate that a discrete fraction of ROCK^{FCC} sediments as a defined tetramer, wherein two antiparallel ROCK dimers likely associate along their coiled-coil axes. This arrangement would permit head-to-tail interactions between the kinase and PH/RBD domains across opposing dimers. We speculate that this higher-order tetramerization stabilizes localized signaling hubs, enhancing avidity toward membrane-associated targets. Consequently, a regulated tetramer-to-dimer transition could serve as as elegant mechanistic switch to amplify ROCK kinase activity at discrete subcellular sites restricting autoinhibitory head-to-tail interactions specifically to the tetrameric state.

3. The manuscript mentions a "stutter" in the heptad repeat of ROCKHR1 and other potential discontinuities in the full coiled coil. The authors should discuss whether these sites act as flexible hinges rather than rigid ruler segments, allowing the kinase domain to reach varied substrates.

Author's response: We thank the reviewer for this insightful suggestion. The observation of a 'stutter' in the heptad repeat of ROCK^{HR1} and other discontinuities throughout the coiled-coil is indeed a key structural feature. We have expanded our Discussion to address the functional implications of these sites. We agree that rather than acting as a strictly rigid 'ruler,' these discontinuities likely serve as molecular hinges or areas of increased local flexibility. This flexibility would allow the long coiled-coil to bend or rotate, providing the kinase domains with the necessary reach and conformational freedom to access diverse substrates in the crowded subcellular environment. This model of a 'flexible tether' is more consistent with ROCK's role in phosphorylating a wide variety of substrates compared to a completely rigid structure. We have updated the manuscript (Line 644-653) to include this discussion on the biological advantage of these coiled-coil discontinuities:

This is reminiscent of ROCK^{SBD}, which features a C-terminal helical bundle where disrupted heptad repeats force adjacent helices into an antiparallel orientation[18]. Rather than acting as a rigid molecular ruler, these periodic discontinuities introduce sites of localized mechanical compliance. These regions act as molecular hinges or universal joints, permitting torsional bending and rotation along the long axis of the coiled-coil. This conformational freedom is likely essential to allow the flanking kinase domains to navigate and access bulky substrates within the crowded cortical environment. Ultimately, obtaining a high-resolution structure of ROCK^{FL} will clarify whether these discontinuities define natural, functionally discrete mechanosensitive subdomains in the core engine.

4. Since HR1 is a known RhoA binding motif in other kinases, it would be valuable to test whether ROCK1's HR1 binds RhoA-GTP, and if so, how? The authors measure RhoA's effect on activity but does not test direct binding to the new HR1 domain. If RhoA fails to bind HR1, that fact itself would be important to report. Some biophysical studies like FRET or SPR to validate would be meaningful.

Author's response: We thank the reviewer for highlighting the importance of the interaction between the HR1 domain and RhoA-GTP. While we agree that this binding is a key regulatory feature, we would like to clarify that we have previously investigated and published this specific interaction. In our earlier work [Blumenstein & Ahmadian et al., 2024], we demonstrated through Fluorsecence assay, that the HR1 domain of ROCK indeed binds RhoA in a strictly GTP-dependent manner. To address the reviewer's concern and ensure the current manuscript is self-contained, we have now explicitly stated this established binding in the Results section (Line 79-83) and added the relevant citation:

Previous fluorescence-based binding assays have demonstrated that the HR1 domain of ROCK binds to RhoA. This finding confirms a direct interaction, which is consistent with the regulatory role of HR1 domains observed in other Rho-associated kinases[7].

Since this interaction has already been biophysically validated in our previous study, we feel that repeating FRET or SPR measurements here would be redundant.

5. From Figure 3D, how do the authors interpret the specificity of the domain of ROC1 interacting with liposome? A quantification of how many ROC1s are engaged to liposome vs free liposome will be useful. Furthermore, correlating that to the pelleting assay can further strengthen this observation

Author's response: We agree that a quantification of the protein-to-liposome stoichiometry would provide additional mechanistical insights in future studies. However, the liposome sedimentation assay used in the present is a widely established and validated method for assessing membrane interactions, as demonstrated in our previous work and several other studies [Akbarzadeh et al. Angew Chem Int Ed Engl. 2022; Mosaddeghzadeh et al. Int J Mol Sci. 2021; Zhang et al. PLoS One. 2014; Mirzaiebadizi, Bazgir et al. Cells, under revision]. We believe that the combination of validated assays and the correlation with our previous publications provides sufficient evidence for the specificity of the ROCK-liposome interaction without the need for additional stoichiometry measurements.

Overall, this is a decent manuscript, where the authors should address the points raised above before it can be accepted.

Author's response: We would like to thank the reviewer for their encouraging assessment of our manuscript and the constructive feedback provided. We have carefully addressed all the points raised, which has significantly helped us to improve the overall quality and clarity of the paper. We hope that the revised version now meets the requirements for acceptance.

Reviewer #3
I co-reviewed this manuscript with one of the reviewers who provided the listed reports. This is part of the Communications Chemistry initiative to facilitate training in peer review and to provide appropriate recognition for Early Career Researchers who co-review manuscripts.

Author's response: We thank you for co-reviewing our manuscript.

Responses to the reviewers' comments on the manuscript COMMSCHEM-26-0138-T:2nd revision

Reviewers' comments:

Reviewer #1:

The manuscript is much improved and almost ready for publication subject to minor corrections. Although I was not provided with a tracked version of the manuscript to be able to see what parts had changed, most of my comments have been addressed. I accept the author's argument about any changes that I requested that have not been made.

Author's response: We thank the reviewer for the positive and encouraging assessment of our revised manuscript. We are grateful that the reviewer finds the manuscript substantially improved and that most of the comments have been satisfactorily addressed. We also appreciate the reviewer's understanding and acceptance of our rationale regarding the requested changes that were not incorporated.
Below, we have carefully addressed the remaining minor corrections and believe that the revised manuscript is now significantly improved and suitable for publication.

Two issues remain:
1. My understanding is that SEC-MALS gives an absolute mass based on the refractive index, independent of the shape or folding of the protein. In that sense it is less reliant on fitting to a model than AUC. In the added text (line 341) it is suggested that the unfolded nature of PKN1 HR1 means that the MALS-based molecular mass is incorrect because of partial unfolding. Can the authors please provide a reference for this statement given that it contradicts the accepted view about MALS?

Author's response: We thank the reviewer for pointing this out and agree that our original statement was incorrect. We agree with the reviewer that SEC-MALS determines the absolute molecular mass from the light-scattering signal and the protein concentration determined from the refractive-index signal. Our interpretation that the low $PKN1^{HR1}$ molecular mass resulted from unfolding-related inaccuracies in the MALS measurement was, therefore, misleading. We have revised the manuscript accordingly and deleted that statement.

Revised manuscript text (Line 333-336): SEC-MALS analysis yielded a molecular mass of 8.9 kDa for $PKN1^{HR1}$, lower than the theoretical monomeric mass of 11.8 kDa (Fig. 2 and Table 1). SEC-MALS provides an absolute molecular-mass measurement that is independent of the protein's globular shape or conformational state.

Revised manuscript text (Line 339-342): The lower-than-expected molecular mass measured by SEC-MALS is potentially arising from partially degraded or truncated species, resulting in a lower mass averaged across the eluting population.

(Please see also our response to the following point)."

In Figure 2B the shape of the mass line for this protein is very different to that for the masses of the other two proteins shown in the same panel. The mass lines for ROCK and RTKN are humped, whereas that for PKN1 is sloping downwards from left to right, implying that the peak is a mixture of species and that the mass cannot be accurately determined.

Author's response: We thank the reviewer for this important observation and agree that the molar-mass trace/line of $PKN1^{HR1}$ differs from those of $ROCK^{HR1}$ and $RTKN^{HR1}$. Whereas the $ROCK^{HR1}$ and $RTKN^{HR1}$ exhibit relatively consistent molar-mass profiles across their respective elution peaks, the $PKN1^{HR1}$ trace shows a pronounced downward slope from the leading to the trailing edge of the peak. This behaviour is most likely due to molecular heterogeneity within the $PKN1^{HR1}$ preparation or degradation/truncation of a single species. Given that the measured mass is lower than the theoretical mass of full-length $PKN1^{HR1}$, partial degradation or truncation is a plausible explanation for the observed heterogeneity and resulting in a lower mass averaged across the eluting population. Accordingly, we have revised the manuscript to avoid interpreting the 8.9-kDa value as the molecular mass of a single homogeneous species. The reported value should instead be considered representative of the molecular mass averaged across the analysed eluting population.

Revised manuscript text (Line 342-348). In contrast to $ROCK^{HR1}$ and $RTKN^{HR1}$, the molar-mass trace of $PKN1^{HR1}$ exhibited a pronounced downward slope across the elution peak, indicating molecular heterogeneity (Fig. 2B). Given the lower-than-expected molecular mass, this heterogeneity may reflect the presence of full-length protein together with partially degraded or truncated species. Therefore, the SEC-MALS-derived mass for $PKN1^{HR1}$ represents the molecular mass averaged across the analysed eluting population rather than that of a single discrete molecular species.

2. I asked for some explanation for the S values and this was provided at length in the rebuttal letter from the authors. Sorry that this was unclear, I was requesting an explanation in the manuscript. Please can a brief note go into the text? It does not have to be as extensive as the explanation provided, just a simple sentence will be sufficient.

Author's response: We thank the reviewer for the clarification. We apologize for the misunderstanding. Following the reviewer's suggestion, we have now incorporated a brief explanation of the S values directly into the manuscript. We believe this addition provides the necessary context and improves the clarity of the text.

Line-327-331-The similar S-values of $ROCK^{HR1}$, $RTKN^{HR1}$ and $PKN1^{HR1}$ arise because the elongated coiled-coil structure of $ROCK^{HR1}$ and $RTKN^{HR1}$ increases their frictional coefficients, causing the larger $ROCK^{HR1}$ dimer (~31 kDa) and $RTKN^{HR1}$ tetramer (~40 kDa) to sediment at rates comparable to the smaller, more flexible $PKN1^{HR1}$ monomer (~12 kDa) and dimer (~24 kDa), respectively

Reviewer #2

The authors have satisfactorily addressed all the concerns raised and have substantially improved the manuscript. We therefore recommend that the manuscript be accepted for publication without further delay.

Author's response: We thank the reviewer for the positive and encouraging assessment of our manuscript. We greatly appreciate the reviewer's valuable comments and constructive suggestions, which have substantially improved the manuscript's quality and clarity. We are pleased that all concerns have been satisfactorily addressed and are grateful for the recommendation to accept the manuscript for publication.

Reviewer #3:

I co-reviewed this manuscript with one of the reviewers who provided the listed reports. This is part of the Communications Chemistry initiative to facilitate training in peer review and to provide appropriate recognition for Early Career Researchers who co-review manuscripts.

Author's response: We thank the reviewer for the positive assessment and constructive comments, which have helped improve the manuscript. We are grateful that the concerns have been satisfactorily addressed and appreciate the recommendation for publication.
