# Supplementary material for: Structural and functional insights into the p160 Rho-associated coiled-coil-containing protein kinase ROCK
Source: Commun Chem. 2026 Nov 4;9:344. doi: 10.1038/s42004-026-02258-2 (PMC13737125; doi:10.1038/s42004-026-02258-2)
Supplement: Supplementary file 2 [file 42004_2026_2258_MOESM2_ESM.pdf]

Supplementary Materials

Structural and functional insights into the p160 Rho-associated coiled-coil-containing protein kinase ROCK*

Badri Nath Dubey[1,2,&,@], Soheila Rezaei Adariani[1,&,#], Lothar Gremer[1,3,4], Radovan Dvorsky[1], Yan Nie[5,#], Ehsan Amin[1], Jens M. Moll[1], Sicai Zhang[1,#], Sander H.J. Smits[6], Melissa A. Graewert[7], Dieter Willbold[3,4], Georg Groth[8], Luitgard Nagel[4], Sabrina Pospich[5], and Mohammad R. Ahmadian[1@]

[1] Institute of Biochemistry and Molecular Biology II, Medical Faculty and University Hospital Düsseldorf, Heinrich Heine University, Universitätsstraße 1, 40225 Düsseldorf, Germany.
[2] Department of Biosciences and Bioengineering, Indian Institute of Technology Dharwad, Dharwad 580011, Karnataka, India.
[3] Institute of Physical Biology, Heinrich Heine University, Universitätsstraße 1, 40225 Düsseldorf, Germany
[4] Institute of Biological Information Processing, Structural Biochemistry (IBI-7), Research Centre Jülich, Leo-Brandt-Str. 5, 52425 Jülich, Germany.
[5] Department of Structural Biochemistry, Max Planck Institute of Molecular Physiology, Otto Hahnstraße 11, 44227 Dortmund, Germany.
[6] Institute of Biochemistry and Center for Structural Studies, Heinrich Heine University, Universitätsstraße 1, 40225 Düsseldorf, Germany.
[7] European Molecular Biology Laboratory, Notkestraße 85, 22607 Hamburg, Germany.
[8] Institute of Biochemical Plant Physiology, Heinrich Heine University, Universitätsstraße 1, 40225 Düsseldorf, Germany.

[&] Authors contributed equally.
[@] Correspondence: badrinathdubey@iitdh.ac.in; reza.ahmadian@hhu.de
* *Running title*: New structural framework for Rho kinase
[#] Current address: Soheila Rezaei Adariani, Department of Neurosurgery, City of Hope Beckman Research Institute and Medical Center, Duarte, CA, USA; Sicai Zhang, Institute of Future Agriculture, Northwest Agriculture and Forestry University, Yangling, Shaanxi 712100, China; Yan Nie, Shanghai Institute for Advanced Immunochemical Studies, ShanghaiTech University, 201210 Shanghai, China;

Table S1. X-ray data collection and refinement statistics of ROCK$^{HR1(Se\text{-}Met)}$

**Data collection**	
Wavelength (Å)	0.979240
Resolution (Å)	37.0-2.2
Number of observed reflections	116242
Number of unique reflections	30449
Completeness (%)	99.4 (98.6)
Redundancy	3.82 (3.78)
R_{merge}(%)	8.3 (55.2)
I/σ(I)	11.57 (2.83)
Space group	C2221
Unit cell	48.26, 87.07, 148.22 90.00° 90.00° 90.00°
**Phasing**	
Selenium-atom sites	5 (final model 8)
Figure of merit (acentric/centric)	0.69/0.64
Resolution range (Å)	19.92-2.50
**Refinement**	
Resolution	37.0-2.2
R_{work}/R_{free} (%)	21.8/28.9
Number of reflections in test set	1106
Average B-factor (Å^2)	39.0
**Deviations from ideal geometry**	
Bond lengths (Å)	0.010
Bond angles (°)	1.14
Ramachandran plot (%)	99.6

Values in parentheses are for the highest resolution shell (2.3-2.2); R_{free} calculated with 5 % of the data that were not used for refinement.

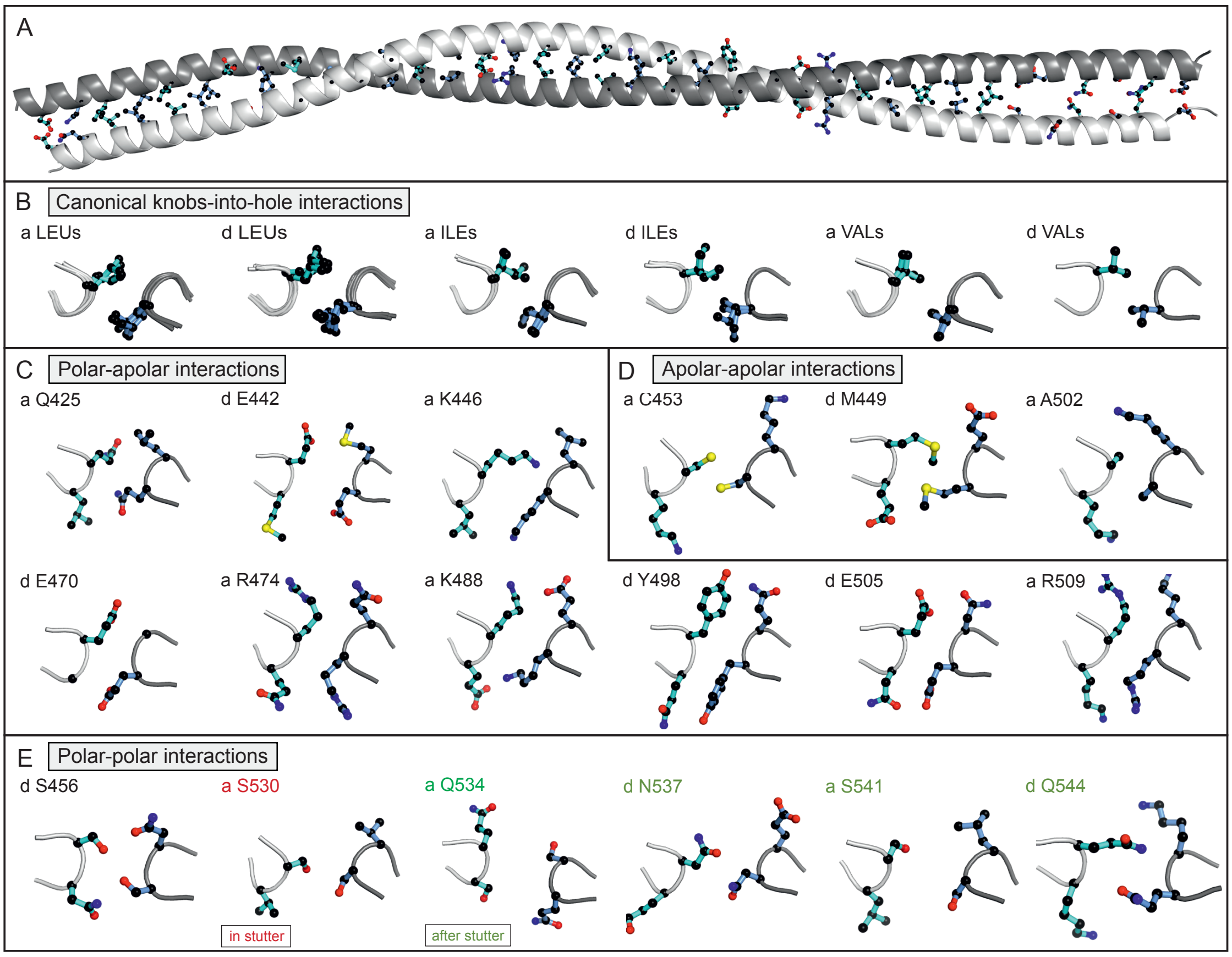

Figure S1. A detailed view of the inter-coiled-coil interactions in ROCK^{HR1}. (**A**) The interacting residues are shown as ball-and-stick models between the ribbon helices of the coiled-coil dimer. Atoms are colored as follows: carbon, black; nitrogen, blue; oxygen, red; sulfur, yellow. (**B–E**) Interhelical interactions are grouped by to their origin. Classical knobs-into-holes packing residues are superimposed for each residue, because this interaction mode is well conserved (**B**). The core interaction of contacts mediated by polar amino acids is hydrophobic (**C–E**). All contacts after the stutter (amino acids 530–533) involve polar amino acids (**E**).

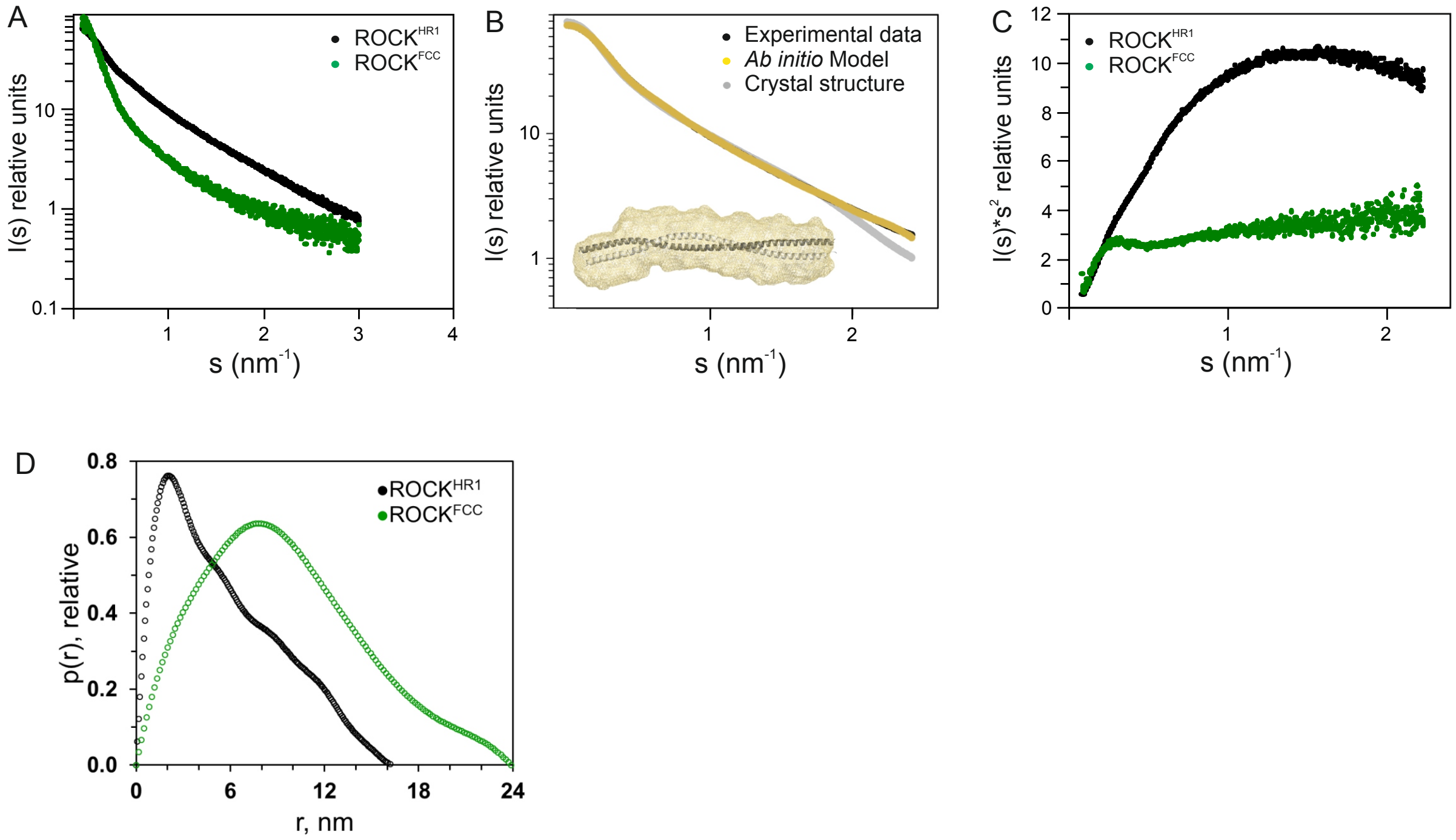

Figure S2. Structural properties of ROCK^{HR1} in solution obtained by small-angle X-ray scattering (SAXS). (**A** The forward scattering intensities were recorded for ROCK^{HR1} (in black) and ROCK^{FCC} (in green). The experimental data are shown as dots. The scattering from *ab initio* models computed by DAMMIF, and the fit from the crystal structure are shown as continuous white lines. The inset shows the ROCK^{HR1} crystal structure superimposed on the averaged ROCK ^{HR1} bead model (in orange), which was calculated using DAMMIF. (**B**) This shows the experimental scattering data, a theoretical scattering pattern calculated from the crystal structure of ROCK^{HR1} using CRYSOL, and a ROCK^{HR1} *ab initio* model calculated from 20 independent DAMMIF runs. The inset shows the SAXS-derived ROCK^{HR1} *ab initio* model superimposed with the ROCK^{HR1} crystal structure. **(C)** Kratky plots of ROCK^{HR1} and ROCK^{FCC}. **(D)** P(r) plots of ROCK^{HR1} and ROCK^{FCC}. All data are summarized in Table 2.

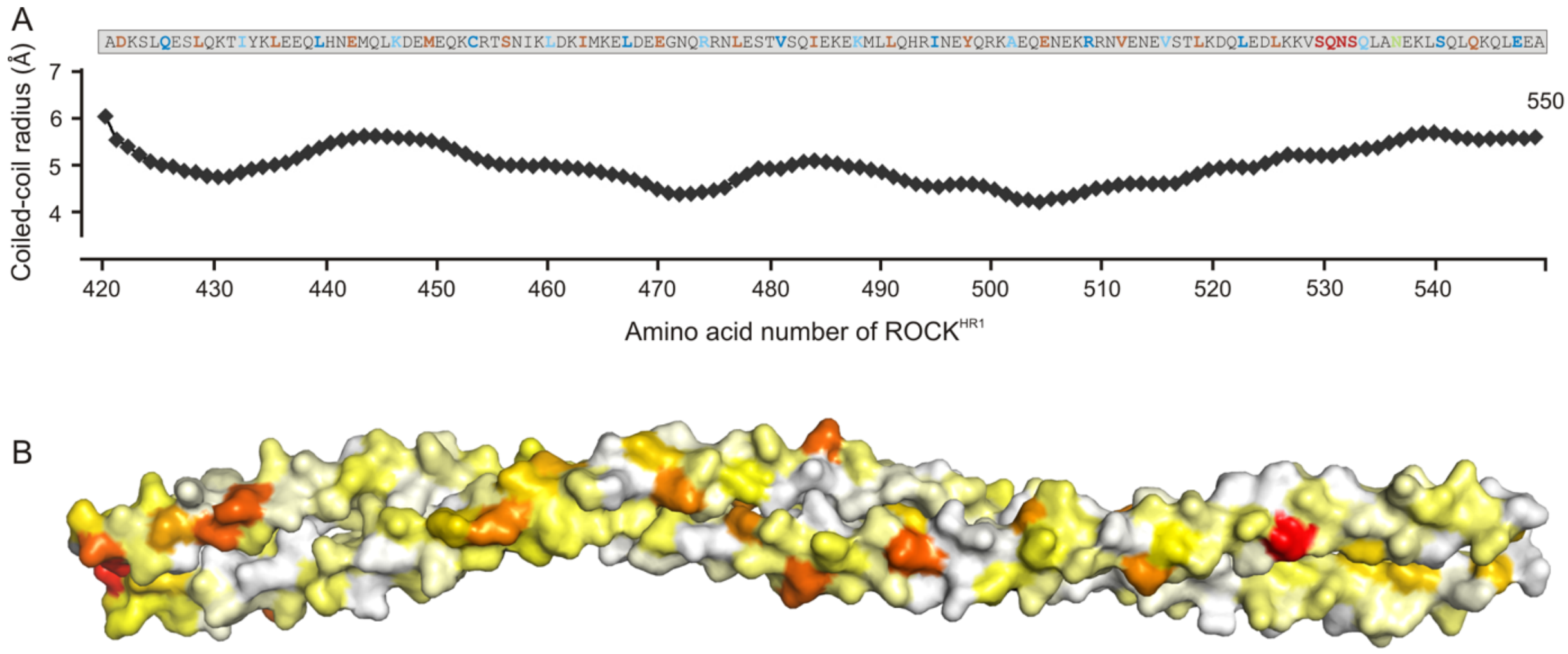

Figure S3. Structural characteristics of ROCK^{HR1} coiled-coil dimer. (**A**) The radius of the coiled-coil is plotted as a function of amino acid number. The amino acid sequence, including the heptad repeats, is shown above. (**B**) The conservation of residues of ROCK^{HR1} is mapped onto the overall surface of the coiled-coil. The color gradient, ranging from white through yellow, orange to red correlates with residue variability, from conserved to most variable.

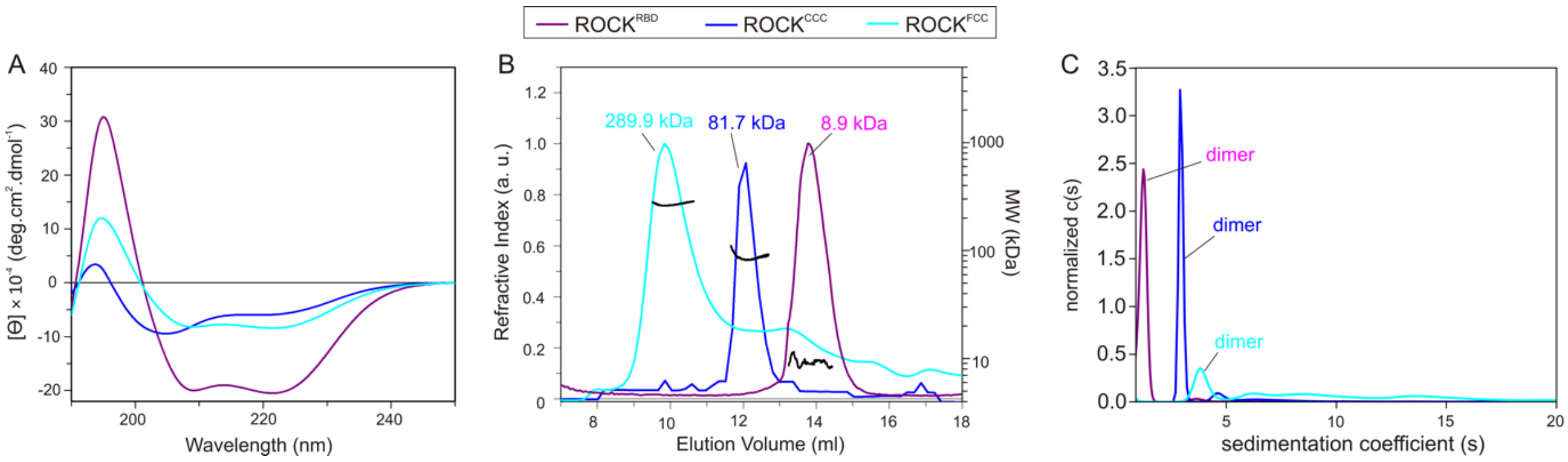

Figure S4. Biophysical properties of ROCK^{RBD}, ROCK^{CCC}, and ROCK^{FCC}. Far-UV CD spectra (**A**), SEC-MALS (**B**), and SV analyses (**C**). Comparison plot of the diffusion-corrected integral sedimentation coefficient distributions obtained from van Holde-Weischet analyses. ROCK^{RBD} is shown in magenta, ROCK^{CCC} in blue, and ROCK^{FCC} in cyan. Analyses were conducted for various fragments of the central amphipathic region of ROCK. All data are summarized in Table 1.

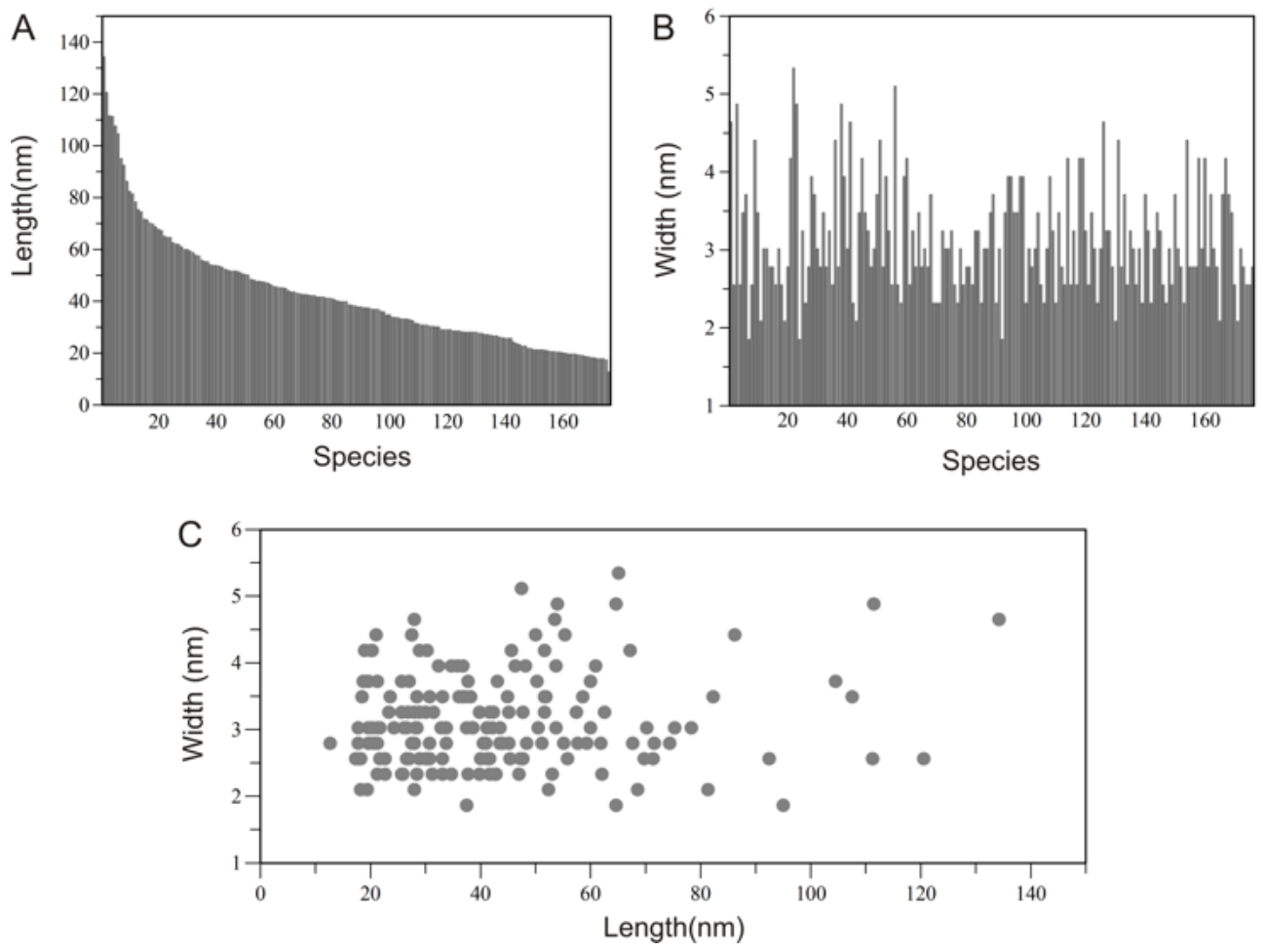

Figure S5. Length and width variations of ROCK^{FCC} molecules. A quantitative analysis of negatively stained ROCK^{FCC} molecules reveals an average length of 42.6 ± 21.7 nm (**A**) and an average width of 3.1 ± 0.7 nm (**B**). The lengths of the rods are ordered from longest to shortest. (**C**) Length-width relationship for all particles.

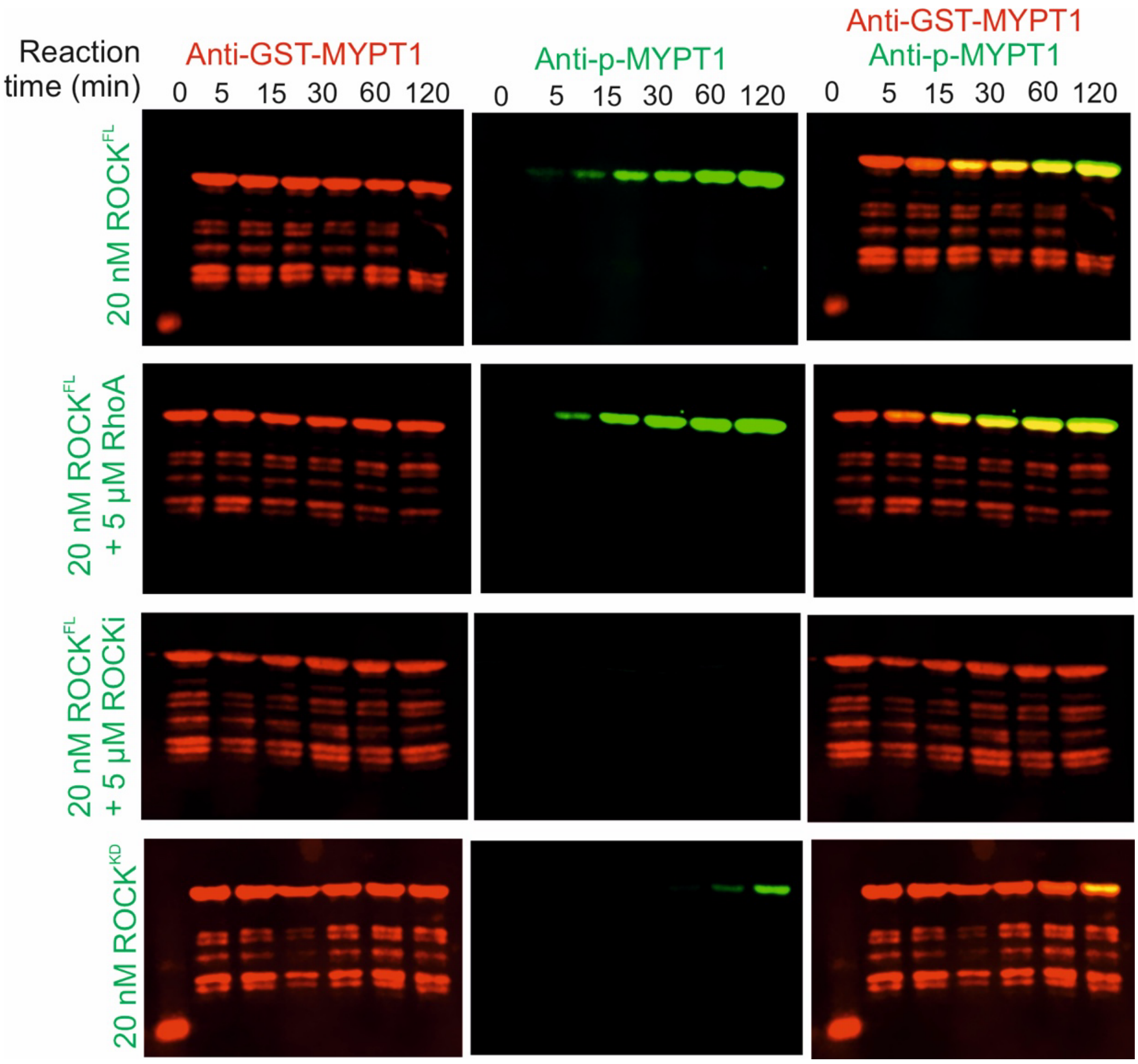

Figure S6. Uncropped blot images are shown in Figure 3E.
